# Cross-Modal Interference-Control Is Reduced in Childhood but Maintained in Aging: A Cohort Study of Stimulus- and Response-Interference in Cross-Modal and Unimodal Stroop Tasks

**DOI:** 10.1037/xhp0000608

**Published:** 2019-04-04

**Authors:** Rebecca J. Hirst, Ella C. Kicks, Harriet A. Allen, Lucy Cragg

**Affiliations:** 1 School of Psychology, University of Nottingham; 2 School of Psychology and Neuroscience, University of St. Andrews; 3 School of Psychology, University of Nottingham

**Keywords:** aging, development, cross-modal distraction, stimulus-interference, response-interference

## Abstract

Interference-control is the ability to exclude distractions and focus on a specific task or stimulus. However, it is currently unclear whether the same interference-control mechanisms underlie the ability to ignore unimodal and cross-modal distractions. In 2 experiments we assessed whether unimodal and cross-modal interference follow similar trajectories in development and aging and occur at similar processing levels. In Experiment 1, 42 children (6–11 years), 31 younger adults (18–25 years) and 32 older adults (60–84 years) identified color rectangles with either written (unimodal) or spoken (cross-modal) distractor-words. Stimuli could be congruent, incongruent but mapped to the same response (stimulus-incongruent), or incongruent and mapped to different responses (response-incongruent); thus, separating interference occurring at early (sensory) and late (response) processing levels. Unimodal interference was worst in childhood and old age; however, older adults maintained the ability to ignore cross-modal distraction. Unimodal but not cross-modal response-interference also reduced accuracy. In Experiment 2 we compared the effect of audition on vision and vice versa in 52 children (6–11 years), 30 young adults (22–33 years) and 30 older adults (60–84 years). As in Experiment 1, older adults maintained the ability to ignore cross-modal distraction arising from either modality, and neither type of cross-modal distraction limited accuracy in adults. However, cross-modal distraction still reduced accuracy in children and children were more slowed by stimulus-interference compared with adults. We conclude that; unimodal and cross-modal interference follow different life span trajectories and differences in stimulus- and response-interference may increase cross-modal distractibility in childhood.

## Public Significance Statement

Children and older adults struggle to inhibit information occurring in the same sense they are focusing on. However, the ability to ignore distractions occurring across the senses is maintained in aging. Thus, although children may experience distractions from all senses, in adulthood certain distractions (such as ignoring a warning light while focusing on the road when driving) may be more distracting than others (ignoring a warning sound while focusing on the road).

Imagine you are reading this article at your favorite café. Suddenly a group sit at the next table and begin chatting loudly. Worse, they are talking about the very topic you are reading about. The ability to ignore this distracting auditory information (conversation) while you focus on visual information (this article) is known as cross-modal interference-control. Despite the every-day occurrence of this “cross-modal” interference, previous research has held a unimodal focus.

Interference (inhibitory) control, is a component of executive function (Miyake et al., 2000). It is the process by which we are able to ignore distractions or interfering stimuli to maintain focus on a task or stimulus (Diamond, 2013; Hasher & Zacks, 1979). One classic measure of interference is the Stroop task (MacLeod, 1992). This requires participants to report the ink-color of a color-word that is either congruent or incongruent with the ink in which it is written (e.g., “RED” in red ink or “RED” in blue ink). Participants are typically slower and less accurate during incongruent verses congruent trials (Stroop, 1935). In the real world, distractions can be either unimodal (chatter in the café while you are also listening to your friend) or cross-modal (chatter while you are reading). However, whether unimodal and cross-modal interference are controlled by the same, or different, mechanisms remains unclear.

Some evidence suggests similar neural mechanisms are recruited for unimodal and cross-modal interference-control. For example, the dorsolateral-prefrontal cortex and anterior cingulate cortex have been implicated in both unimodal (Botvinick, Braver, Barch, Carter, & Cohen, 2001; Silton et al., 2010) and cross-modal (Weissman, Warner, & Woldorff, 2004) control. Nevertheless, behavioral evidence suggests unimodal and cross-modal Stroop effects manifest themselves differently. Cross-modal Stroop effects appear smaller than the traditional Stroop effect. Furthermore while the traditional Stroop effect increases with response time latency, cross-modal interference appears equivalent across the response time distribution (Elliott et al., 2014); suggesting a different locus of interference. Here we extend these findings by comparing whether unimodal and cross-modal interference manifest a similar pattern of development and decline, and whether unimodal and cross-modal interference occur at similar points in the information-processing stream.

## Interference in Development and Aging

Our ability to suppress interference from irrelevant information is proposed to improve with development and deteriorate with age (Comalli, Wapner, & Werner, 1962). In a life span study of the unimodal Stroop effect, Comalli et al. (1962) found interference was greatest in children aged 7–8 years and adults aged over 60. If interference-control is domain general, this trajectory should generalize to cross-modal Stroop tasks. Nevertheless, aging does not reflect development in reverse, and different factors likely contribute toward unimodal and cross-modal control. For example, while unimodal Stroop tasks entail inhibition of written words, cross-modal tasks entail inhibition of speech processing. It is likely that these facets of cognition are differentially affected by development and aging. As such, it cannot be assumed that cross-modal interference matches unimodal interference.

Increased susceptibility to distraction in childhood has been attributed to the protracted development of frontal brain networks recruited for top-down control over behavior (Fuster, 2002; Olesen, Macoveanu, Tegnér, & Klingberg, 2007). In line with the recruitment of prefrontal regions, such as the anterior cingulate cortex, in Stroop performance (Laird et al., 2005) it has consistently been shown that children manifest increased Stroop interference (Bub, Masson, & Lalonde, 2006; Wright, 2017). Although increased cross-modal Stroop interference in childhood has been reported (Hanauer & Brooks, 2003, 2005; Thomas, Nardini, & Mareschal, 2017) these effects have obtained less research focus. Moreover, because these studies did not include unimodal comparison tasks, a direct comparison of unimodal and cross-modal Stroop effects has not yet been possible. It has been shown that poorer attentional resources in early childhood can paradoxically reduce distractibility to multisensory stimuli in childhood (Matusz et al., 2015). Thus, the impact of multisensory distraction in childhood requires further investigation.

There has not been a study of cross-modal Stroop effects in adults older than 40 years of age. However, in a thorough review of age-related distraction, Guerreiro, Murphy, and Van Gerven (2010) suggested that although older adults typically show enhanced interference in unimodal tasks, cross-modal interference appears equivalent across older and younger adults, particularly if irrelevant information is auditory (Guerreiro, Adam, & Van Gerven, 2014; Guerreiro, Anguera, Mishra, Van Gerven, & Gazzaley, 2014; Guerreiro, Murphy, & Van Gerven, 2013). This was found for studies using the irrelevant sounds task (Bell & Buchner, 2007; Belleville, Rouleau, Van der Linden, & Collette, 2003), the cross-modal Simon task (Proctor, Pick, Vu, & Anderson, 2005) and studies assessing memory for irrelevant auditory information (Murphy, McDowd, & Wilcox, 1999). As many of these studies used visual tasks with verbal distractors, older adults might be expected to be able to ignore spoken words while focusing on visually presented color information in the cross-modal Stroop task.

On the other hand, older adults have been shown to benefit from cross-modal congruency more than younger adults. Laurienti, Burdette, Maldjian, and Wallace (2006) found that older adults responded faster to colored circles when presented alongside a congruent spoken color-word. This benefit was greater than presenting circles with a written color-word and greater in older versus younger adults. In contrast to findings showing older adults effectively “filter out” auditory information while focusing on vision (Bell & Buchner, 2007; Belleville et al., 2003; Murphy et al., 1999; Proctor et al., 2005) this suggests stronger cross-modal Stroop effects in older adults.

## Sensory Processing in Development and Aging

When considering changes in unimodal compared with cross-modal Stroop performance for participants of different ages it is important to also consider the effects of development and aging on the auditory and visual sensory systems and how information from different senses is integrated. Stroop interference has been attributed to an asymmetry in the ease of access to word and color information, whereby color naming is more difficult than word reading, as it requires an intermediate processes to retrieve the word to be spoken (Melara & Algom, 2003; Roelofs, 2005). Many factors change the accessibility of color and word information in aging. Deteriorations in color vision may limit the accessibility of color; thus, increasing Stroop interference in older adults (Anstey, Dain, Andrews, & Drobny, 2002; Ben-David & Schneider, 2009, 2010; Cooper, Ward, Gowland, & McIntosh, 1991). Alternatively, age related hearing loss might make auditory distractors in cross-modal Stroop tasks less salient. Thus, to conclude that older adults have maintained ability to ignore cross-modal distractions, as proposed by Guerreiro et al. (2010), it is essential to ensure distractors are presented well above perceptual thresholds for all age groups being tested. Some studies using simple stimuli have attempted to control for the intensity of auditory distractors by presenting irrelevant sounds based on participants’ thresholds (Belleville et al., 2003). Studies using spatial cueing tasks have also matched response times to auditory and visual stimuli across age groups (Guerreiro, Adam, & Van Gerven, 2012) and studies assessing cross-modal interference in memory for faces and voices have adjusted stimuli to a “comfortable” level to control for individual differences (Guerreiro, Anguera, et al., 2014; Guerreiro, Eck, Moerel, Evers, & Van Gerven, 2015). However, to our knowledge, no study has yet attempted to equate visual and auditory stimulus intensity based on individual sensory thresholds, which may provide a more precise approach to equating stimuli.

It is also important to consider the role that age-related changes in multisensory integration may have on cross-modal interference. The neural processes underlying multisensory integration are thought to develop late in humans (Burr & Gori, 2012; Ernst, 2008) and are susceptible to plasticity depending on early sensory experience (Carriere et al., 2007; Polley et al., 2008). In line with the protracted development of the visual cortex (Graven & Browne, 2008b) relative to the auditory cortex (Graven & Browne, 2008a), children under 10 years of age appear less susceptible to multisensory illusions in which vision alters auditory perception (Hirst, Cragg, & Allen, 2018; Hirst, Stacey, Cragg, Stacey, & Allen, 2018; Tremblay et al., 2007) and more susceptible to illusions in which audition changes vision (Innes-Brown et al., 2011). However, these effects are modulated by early sensory experience (Narinesingh, Goltz, Raashid, & Wong, 2015; Schorr, Fox, van Wassenhove, & Knudsen, 2005). Given this, children might be more susceptible to interference from audition when focusing on vision than vice versa and this may be influenced by experience. Furthermore, children under 11 years of age show lower audio-visual facilitation of response times (Barutchu, Crewther, & Crewther, 2009; Barutchu et al., 2010) and the time window over which auditory and visual information are integrated narrows between the ages of 4 and 6 and continues to narrow into adulthood (Lewkowicz & Flom, 2014; Noel, De Niear, Van der Burg, & Wallace, 2016). As such, we would expect general developmental differences in response times to audio-visual stimuli as well as the extent to which auditory and visual stimuli are attributed to the same object.

Findings regarding the effect of aging on multisensory integration have been mixed (Brooks, Chan, Anderson, & Mckendrick, 2018). Some findings suggest enhanced multisensory integration in aging: Older adults appear more susceptible to multisensory illusions in which vision modulates audition (Sekiyama, Soshi, & Sakamoto, 2014) and vice versa (DeLoss, Pierce, & Andersen, 2013; Noel et al., 2016; Parker & Robinson, 2018), and manifest greater multisensory enhancement of response times (Laurienti et al., 2006). Older adults have been shown to integrate information over wider (Bedard & Barnett-Cowan, 2016; Chan, Pianta, & Mckendrick, 2014; Noel et al., 2016) and similar (Bedard & Barnett-Cowan, 2016) time windows compared with younger adults depending on the task. A recent review of multisensory processing in aging highlighted the need to consider unisensory change when assessing multisensory integration in aging (Brooks et al., 2018). As such, within the current paper we control for differences in sensory ability while measuring cross-modal effects.

## The Locus of Interference

Irrelevant information can interfere at all stages of the information-processing stream. This includes early stages of processing at the level of encoding; stimulus-interference, and later processing at the level of response selection; response-interference (Chen, Bailey, Tiernan, & West, 2011; Cragg, 2016; De Houwer, 2003; Jongen & Jonkman, 2008; Killikelly & Szűcs, 2010, 2013; Zhang & Kornblum, 1998). In traditional interference tasks however, stimulus- and response-interference are confounded. Incongruent conditions require participants to encode two conflicting perceptual representations and select from two competing responses, while congruent conditions prime complementary perceptual representations and require the same response.

To separate these processes, De Houwer (2003) presented participants with a Stroop task in which two colors were mapped to a left button and two to a right button. Thus, the color-word and ink-color could be congruent, incongruent but mapped to the same response (stimulus-incongruent), or incongruent and mapped to different responses (response-incongruent). Using the De Houwer (2003) paradigm, it is possible to separate three types of interference. General interference, encompassing both stimulus- and response-interference, which can be calculated as:

Generalinterference=ResponseIncongruent/Congruent 1

where “Response Incongruent” reflects response time, or accuracy, under response incongruent conditions and “Congruent” reflects response time, or accuracy under congruent conditions.

Following this, stimulus- and response-interference can be isolated as:

StimulusInterference=StimulusIncongruent/Congruent 2

ResponseInterference=ResponseIncongruent/StimulusIncongruent 3

Thus, response-interference reflects *additional* interference occurring because of the response demands of the task (i.e., over and above interference arising from stimulus level competition). General interference, thus, reflects the sum of stimulus- and response-interference.

Stimulus- and response-interference are candidate measures to tease apart the mechanisms underlying unimodal and cross-modal interference. For instance, Chen et al. (2011) used a variant of the De Houwer (2003) paradigm in which participants were shown six color words in the same or different colored ink. Three colors were mapped to one button while three were mapped to the other (thus, producing congruent, stimulus-incongruent, and response-incongruent conditions). To map when different types of interference occurred, stimulus- and response-interference were plotted as a function of response time. Response-interference was found to occur at longer response latencies while stimulus-interference remained relatively constant across the response time distribution (Chen et al., 2011). These findings parallel the differences between unimodal and cross-modal interference reported by Elliott et al. (2014) and perhaps indicate unimodal and cross-modal interference to arise from different types of interference (stimulus- compared with response-interference).

The balance of stimulus- and response-interference has also been shown to shift in midchildhood, between the ages of 7 and 10 years (Cragg, 2016), and shift from young to midadulthood between the ages of 30 and 45 years (Killikelly & Szűcs, 2013). Cragg (2016) found that 7-year-olds children showed greater stimulus-interference while 10-year-olds and adults showed greater response-interference. Killikelly and Szűcs (2013) report increased stimulus-interference in adults aged >40 years. If unimodal and cross-modal interference share associated mechanisms we would expect this shift to also occur under cross-modal conditions.

## The Current Study

Comparing developmental trajectories in childhood and old age, and examining the point in the information-processing stream at which interference occurs, can help to elucidate whether similar mechanisms underpin unimodal and cross-modal interference-control. However, to our knowledge no previous study has attempted to separate stimulus- and response-interference within a cross-modal paradigm and explore these processes in both development and aging. Here we report the findings from two experiments which used adapted versions of the color-word Stroop paradigm to investigate (a) whether unimodal and cross-modal interference follow similar patterns of development and deterioration; (b) if stimulus- and response-interference contribute to cross-modal, as well as unimodal, interference; and (c) whether the relative contribution of stimulus- and response-interference under unimodal and cross-modal conditions changes across the life span. In Experiment 1 we compared the ability to focus on vision and ignore either visual (unimodal) or auditory (cross-modal) information. In Experiment 2 we compared whether a similar pattern of effects occur when focusing on audition and ignoring vision to explore whether findings generalized across cross-modal conditions. In both experiments, we focus on age groups in which multisensory and interference control processes are known to be immature; below 11 years (Hirst, Stacey, et al., 2018; Noel et al., 2016), and susceptible to age-related decline; above 64 years (Comalli et al., 1962; Noel et al., 2016). More important, in both experiments we also controlled for sensory differences that may differentially affect the balance of relevant and irrelevant information between age groups. This was achieved by ensuring distractors were presented at equivalent levels above perceptual thresholds for all participants. The findings from this comprehensive study will help to understand whether similar mechanisms underpin unimodal and cross-modal interference-control and whether such mechanisms are equally susceptible to developmental maturation and aging.

## Experiment 1

In Experiment 1 we compared unimodal and cross-modal interference in children, young adults and older adults using a modified version of the color-word Stroop paradigm. We measured participants’ ability to ignore written words (unimodal interference) and spoken words (cross-modal interference) while naming colored rectangles. If similar processes underlie unimodal and cross-modal interference-control we would expect unimodal and cross-modal interference to both manifest U-shape trajectories across children, young adults and older adults, with greater interference in children and older adults compared with young adults. Furthermore, we would predict stimulus- and response-interference to be evident under unimodal and cross-modal conditions, and manifest similar patterns across the response time distribution (Chen et al., 2011; Killikelly & Szűcs, 2010).

### Method

#### Participants

Appropriate sample sizes for adult samples were estimated a priori via a power analyses in G*power 3.1 (Faul, Erdfelder, Lang, & Buchner, 2007) using a Cohens effect size of 0.5. This was assumed based upon well documented unimodal Stroop effects in young adults, and greater unimodal Stroop effects in children and older adults (Comalli et al., 1962; MacLeod, 1991) while considering the limited cross-modal Stroop effects literature. We calculated the sample size required to detect a Within × Between interaction with three groups and four measurements. Thus, the sample size was large enough to detect a difference between unimodal and cross-modal stimulus and response-interference between age groups (i.e., a 2 [sensory condition: unimodal vs. cross-modal] × 2 [interference type: stimulus vs. response-interference] × 3 [age group] mixed analysis of variance [ANOVA]). The exact parameters used within the power analysis were, therefore; a “Repeated measures within-between interaction,” α = .05, power = .95, number of groups = 3, number of measures = 4, nonsphericity correction = 1. This analysis indicated a need for a minimum of 29 participants per age group (87 in total), a criterion that was met by all three samples. Sample size for children was opportunistic, data were gathered at a public engagement event and all children attending the event had the opportunity to participate.

Thirty-three young adults (mean age 22.44 years, range 18–25, 23 female), 39 older adults (mean age 71.25 years, range 61–85, 23 female) and 49 children (mean age 9.03 years, range 6–11, 21 female) took part. Young adults were staff and students at the University of Nottingham that were known to the researchers or recruited via the university’s research participation scheme. Older adults were healthy participants recruited via the university’s volunteer register and children were recruited via Summer Scientist Week, a public engagement event at the university (www.summerscientist.org). In exchange for participation, young adults were offered credit as part of their degree, older adults were paid £7 and children received “tokens” to be spent on games at the event.

Two older adults were excluded from later analysis, one because of hearing aid use and one because of red-green color blindness. Four children were excluded from later analysis because of parents reporting diagnosed developmental disorders. Following these exclusion criteria and the exclusion of outliers (see analysis) this left a final sample of 42 children, 31 young adults and 32 older adults for analysis.

#### Equipment

Visual stimuli were presented via a Mac mini 3.1 on a 16” KFC Smile CA6748SL CRT monitor (resolution 1024 × 768@85Hz) calibrated via Psychopy (Peirce, 2007, 2009) using a PR-655 Spectrascan. An adapted “noisy bit” (Hirst & Allen, 2018) method was used to present low contrast stimuli (Allard & Faubert, 2008; Pelli & Zhang, 1991). Stimuli were always presented at a viewing distance of ∼57 cm maintained via a chinrest. Auditory stimuli were presented via Senheiser HMD280 PRO headphones calibrated using a Sinus Apollo and Sinus Samuri v2.26 software using a Bruel and Kjaer 4153 (Naerum, Denmark) artificial ear, with 4134 ½” microphone (to BS EN 60,318–1:2009), and Bruel and Kjaer 4157 Ear simulator, with 4134 ½” microphone (to IEC 711–1981, ANSI S3.25–1979 [R 1986]).

#### Stimuli

Notably, in the traditional Stroop task, relevant (color) and irrelevant (word) dimensions are part of the same object (a color word written in colored ink). However, under cross-modal conditions the visibly seen color is not an attribute of the written word, rather the spoken word is separate from the visually presented color. This may result in reduced Stroop interference, as color and word information are not processed as part of the same object and Stroop interference has been shown to be larger when color and word information are integrated (Macleod, 1998). The current study used a color patch Stroop to alleviate this imbalance between unimodal and cross-modal conditions.^1^ Visual stimuli consisted of four colored rectangles (initial luminance without overlaid word stimuli: red = 24.47cd/m^2^, green = 87.45cd/m^2^, blue = 16cd/m^2^, yellow = 109.3cd/m^2^; RGB color space: red = [255, 0, 0], green = [0, 255, 0], blue = [0, 0, 255], yellow = [255, 255, 0]). Rectangles were presented with one of five written or spoken color words “RED,” “GREEN,” “BLUE,” “YELLOW,” or “BROWN.” Written color-words were presented in black font.

To prevent participants looking at “blank” colored areas of stimuli (thereby allowing the task to be performed without reading the words), the dimensions of the rectangle varied depending on the word it was presented with (“RED” = 0.40 × 0.15°, “YELLOW” = 0.65 × 0.15°, “BLUE” = 0.45 × 0.15°, “GREEN” = 0.55 × 0.15°, “BROWN” = 0.60 × 0.15°). When deriving participants’ thresholds before Stroop performance (see online supplementary material) the dimensions of the rectangle were always set to 0.65 × 0.15° to avoid associative learning between rectangle size and word to be identified.

Spoken color-words were spoken in a female voice with an average duration of 478.2 ms (“RED” = 441 ms, “GREEN” = 501 ms, “BLUE” = 409 ms, “YELLOW” = 485 ms, “BROWN” = 555 ms). By presenting auditory stimuli binaurally while participants fixated upon stimuli we assume visual and auditory stimuli were colocalized to the same location (Stern, Brown, & Wang, 2006). To control for extraneous noise, Brown noise was presented alongside all auditory stimuli. Brown noise was created via Audacity (Version 2.0.6.0) and set to 60 dB throughout threshold and Stroop tasks.

Before performance of the Stroop task, thresholds (contrast/volume required for participants to identify visual/spoken words on 79% of trials) were measured (see online supplementary material). Written words and spoken words were then presented 10× (20 dB) above threshold. For visual stimuli, if this value fell above 100% opacity then stimuli were presented at 100% opacity. For auditory stimuli, if this value was higher than 65 dB then stimuli were presented at 65 dB. Auditory stimuli had to be presented at maximum for 5 young adults, 17 older adults (4 of which were later removed as outliers and one of which was removed because of difficulty deriving an auditory threshold) and 11 children (2 of which were later removed as outliers). Notably, auditory stimuli were still set well above threshold, and were, therefore, audible, for all of these participants (*M* = 13.67 dB above auditory threshold; range = 5.5–19.5 dB above auditory threshold). No participants required visual stimuli to be set to maximum.

##### Balancing sensory information

The mere presence of a stimulus in one sensory modality can affect participants’ detection of stimuli in another (e.g., Frassinetti, Bolognini, & Làdavas, 2002), response times to stimuli (Gondan, Niederhaus, Rösler, & Röder, 2005) and influence response times differently across age groups (Diederich, Colonius, & Schomburg, 2008; Laurienti et al., 2006). Thus, we balanced sensory input across unimodal conditions and cross-modal conditions by presenting auditory “babble” (multiple speakers saying different words at once) during unimodal conditions and visual babble (multiple words overlaying one another) during cross-modal conditions. Thus, auditory and visual information was presented in both unimodal and cross-modal conditions.

Auditory babble consisted of 96 unique samples of three-speaker babble, created using three words from different speakers and jittering word onset and offset. Words were noncolor-word nouns, speakers were selected from 12 speakers (six female) and babble duration was matched to the average duration of spoken word stimuli.

Visual babble consisted of 96 unique samples of three-word babble, created from three overlaid words with jittered onset/offset. The same words were used to create visual and auditory babble. Visual babble varied in length to approximately match the length of written color words (19 samples of red, green, blue and yellow length and 20 samples for brown length). When deriving thresholds, visual and auditory babble were restricted to the same length/duration. These restrictions were implemented to prevent associative learning between auditory/visual babble-length and color-word to be identified. In the Stroop task babble stimuli were set to appear 10× (20 dB) above each participant’s visual or auditory threshold (with a maximum of 100% opacity/65 dB) as above.

### Procedure

**Figure 1 fig1:**
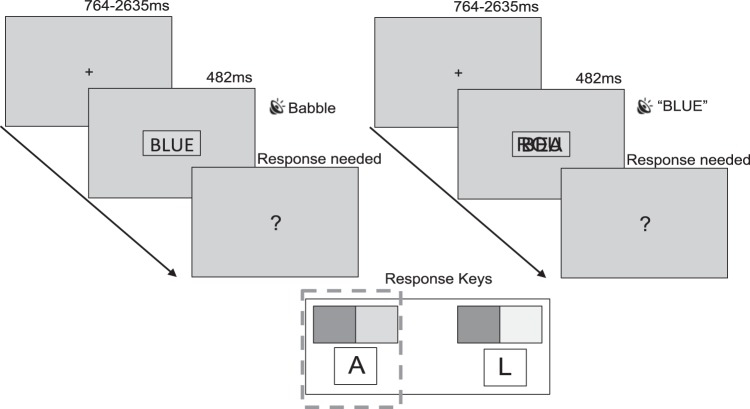
Schematic illustrating unimodal (left) and cross-modal (right) protocol. Participants fixated for 764–2,635 ms. Under unimodal conditions participants were then presented with a colored rectangle containing a written color-word alongside a sample of auditory babble embedded in 60 dB Brown noise. Under cross-modal conditions participants were presented with a colored rectangle overlaid by a sample of written babble alongside a spoken color-word embedded in 60 dB Brown noise. Participants were asked to identify the color of the rectangle as fast and accurately as possible. If no response had been made after stimulus offset a question mark was presented, signaling the need for a response. The dashed box indicates that in this example a left button press was needed.

All participants completed the Stroop procedure illustrated in Figure 1. Participants were instructed to sort colored “tickets” (rectangles) into two boxes using two buttons (the “A” and “L” keys on a QWERTY keyboard), one for red/green rectangles and the other for blue/yellow rectangles (response mappings were counterbalanced across participants).[Fig-anchor fig1]

On each trial a fixation point was presented for 764–2,635 ms. After this, a colored rectangle overlaid by a written word (in unimodal conditions) or visual babble (in cross-modal conditions) was presented for 482 ms. Visual stimuli were presented simultaneously with auditory babble (in unimodal conditions) or a spoken color word (in cross-modal conditions) embedded in 60 dB Brown noise. Participants identified the color of the rectangle by pressing the left or right key and were instructed to ignore any written or spoken information. Participants were told they could respond as soon as they saw the ticket and to be as fast and accurate as possible. If no response had been made after stimulus offset a question mark was presented, signaling the need for a response.

In addition to sensory condition (unimodal vs. cross-modal) there were four levels of congruency; congruent (i.e., “RED” with a red rectangle), incongruent but mapped to the same response; stimulus-incongruent (i.e., “GREEN” with a red rectangle), incongruent and mapped to a different response; response-incongruent (i.e., “BLUE” with a red rectangle), or incongruent and mapped to no response; “neutral” (i.e., “BROWN” with a red rectangle).

Participants completed the unimodal and cross-modal conditions in separate blocks (counterbalanced across participants) each containing 96 trials (24 per condition). An optional break was offered after 48 trials. A 20 trial practice block was given before the first block and a 10 trial practice block before the second block to accustom participants to the new stimuli. Trials were presented in a pseudorandomized order for each participant such that no immediate repeats in the color rectangle or color-word would occur and congruency condition would not be repeated more than twice.

### Analysis and Results

We aimed to answer the following two questions:

1 Does general interference differ between unimodal and cross-modal conditions, and is this different between age groups?
2 Can any differences between unimodal and cross-modal interference be explained by comparing stimulus- and response-interference?

To answer these questions we defined general interference, stimulus- and response-interference ratios as noted in Eqs. 1, 2 and 3. The use of ratio scores allows for comparison of interference while controlling for general differences in speed across age groups. However, we also provide a full analysis of raw data (before calculation of ratios) in online supplementary material (section S3). These supplementary analyses are consistent with the currently reported findings, in which we use ratio scores throughout. Additionally, analysis of participants’ thresholds and a description of the protocol used to derive thresholds can be found in supplementary sections S2 and S1, respectively. It should be noted that, to remain conservative, throughout our analyses we discuss *p* values ≤ .01 as significant, however, all *p* values are reported throughout.^2^ All data from this study are available via https://osf.io/vmdz9/.

Outliers were removed from each participants’ data by identifying response times that fell outside the range of the absolute deviation around the median (Leys, Ley, Klein, Bernard, & Licata, 2013). Outlying participants were identified and removed by calculating the mahalanobis distance of each subject from the χ^2^ distribution of their age group based upon response time and accuracy during congruent, stimulus-incongruent and response-incongruent trials under unimodal and cross-modal conditions. Cases holding a probability of <.001 of belonging to the population were removed from analyses. This resulted in 9 participants being removed (3 children, 2 young adults, and 4 older adults). One older adult was also removed because of difficulty deriving an appropriate auditory threshold.

#### General interference

**Figure 2 fig2:**
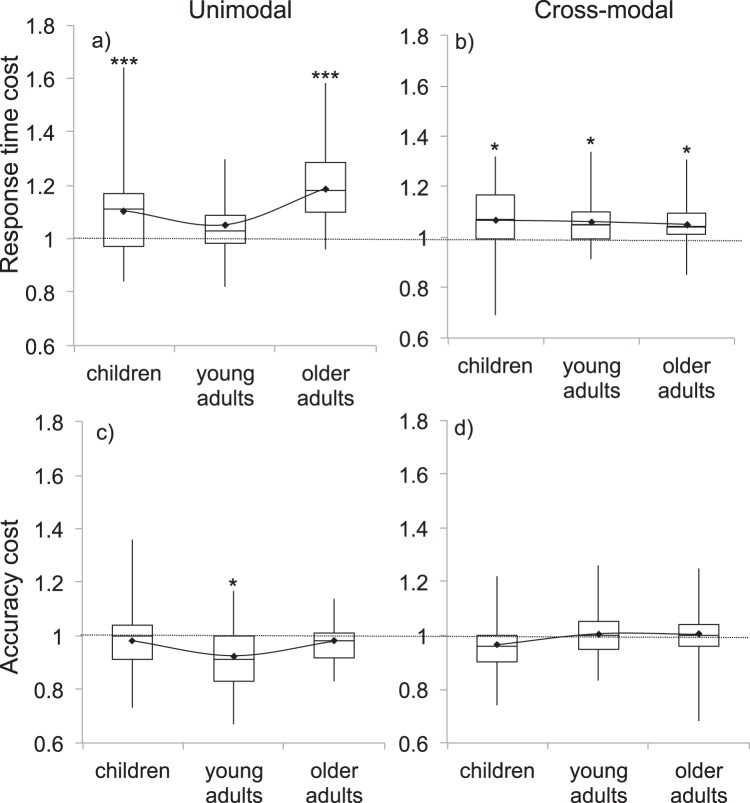
General interference under unimodal (left) and cross-modal (right) conditions in terms of response time (top) and accuracy (bottom). Response time ratios greater than 1 indicate slowing. Accuracy ratios less than 1 indicate accuracy decrements. Black diamonds indicate means used for analyses. The line connecting means demonstrates the extent of the quadratic (U-shape) trajectory. Asterisks indicate *t* tests comparing ratio to 1 (*.05, **.01, ***.001) Bonferroni corrected for six comparisons.

Response time and accuracy data were submitted to two separate 2 (sensory condition) × 3 (age group) ANOVAs to compare unimodal to cross-modal interference. Significant interactions were followed up with simple main effects analyses adjusted for multiple comparisons via Bonferroni correction. Significant main effects of age group were followed with post hoc quadratic tests to examine whether each interference type followed a U-shape trajectory across age groups. Across analyses, a series of Bonferroni corrected *t* tests were also performed to compare ratio scores to 1. This provides a comparison of each interference score relative to baseline. The results of these comparisons are indicated within each figure. Results for response times and accuracy are reported below and shown in Figure 2.[Fig-anchor fig2]

##### Response times

The effect of age group on response time ratios failed to reach our conservative criterion for significance (*F*(2, 102) = 3.17, *p* = .05, η_*p*_^2^ = .06). A significant effect of sensory condition (*F*(1, 102) = 9.51, *p* = .003, η_*p*_^2^ = .09) occurred, which interacted with age group (*F*(2, 102) = 5.26, *p* = .007, η_*p*_^2^ = .09). Although general interference was higher (i.e., caused more slowing) under unimodal compared with cross-modal conditions, this arose because of a significant difference in older adults (*p* < .001), which was not present in young adults (*p* = .77) or children (*p* = .16). A quadratic test indicated a significant U-shape function under unimodal conditions (*F*(1, 102) = 8.58, *p* = .004, η_*p*_^2^ = .08) that did not occur under cross-modal conditions (*F*(1, 102) = .01, *p* = .93, η_*p*_^2^ < .001).

##### Accuracy

There was no main effect of sensory condition on accuracy (*F*(1, 102) = .4.02, *p* = .5, η_*p*_^2^ = .04), no interaction between sensory condition and age (*F*(2, 102) = 3.72, *p* = .03, η_*p*_^2^ = .07) and no main effect of age group (*F*(2, 102) = 1.14, *p* = .32, η_*p*_^2^ = .02). Quadratic trends did not reach significance in either unimodal (*F*(1, 102) = 5.93, *p* = .02, η_*p*_^2^ = .06) or cross-modal (*F*(1, 102) = 1.04, *p* = .31, η_*p*_^2^ = .009) conditions.

#### Stimulus- and response-interference

**Figure 3 fig3:**
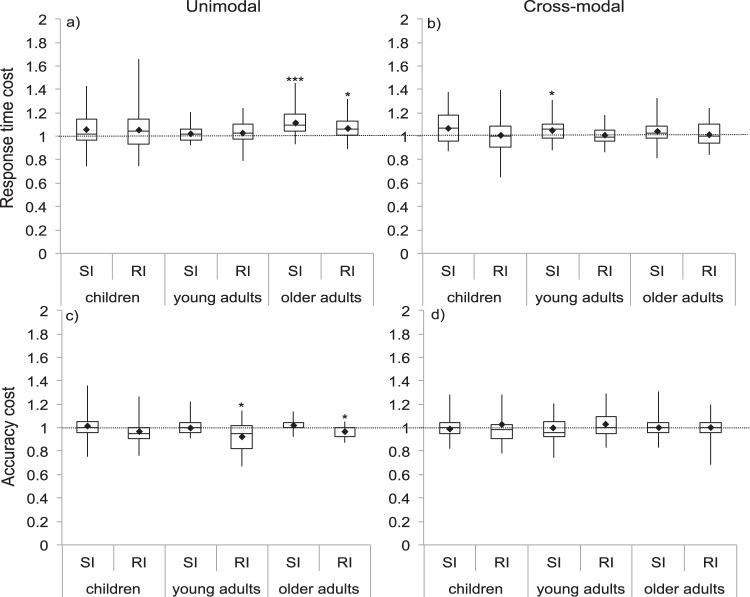
Stimulus (SI) and response (RI) interference ratios in terms of response time (top) and accuracy (bottom) under unimodal (left) and cross-modal (right) conditions. Response time ratios higher than 1 indicate slowing. Accuracy ratios lower than 1 indicates accuracy decrements. Black diamonds indicate means used for analyses. Asterisks indicate *t* tests comparing ratio to 1 (*.05, **.01, ***.001) Bonferroni corrected for 12 comparisons.

To examine differences in the contribution of stimulus- and response-interference under unimodal and cross-modal conditions, stimulus- and response-interference ratios for response time and accuracy were submitted to two separate 2 (sensory condition: unimodal, cross-modal) × 2 (interference type: stimulus-interference, response-interference) × 3 (age group: children, young adults, older adults) ANOVAs. Results for response times and accuracy are reported below and shown in Figure 3.[Fig-anchor fig3]

##### Response times

Unimodal interference was greater than cross modal interference (*F*(1, 102) = 9.34, *p* = .003, η_*p*_^2^ = .08) and this interacted with age group (*F*(2, 102) = 4.86, *p* = .01, η_*p*_^2^ = .08), but did not interact with interference type (*F*(1, 102) = .62, *p* = .54, η_*p*_^2^ = .01). The difference between stimulus- and response-interference did not reach significance (*F*(1, 102) = 4.35, *p* = .04, η_*p*_^2^ = .04) and did not interact with age group (*F*(2, 102) = 0.09, *p* = .92, η_*p*_^2^ = .002) or sensory condition (*F*(1, 102) = 1.36, *p* = .25, η_*p*_^2^ = .01). There was no three-way interaction between sensory condition, age group and interference type (*F*(2, 102) = .62, *p* = .54, η_*p*_^2^ = .01). In line with analyses of general interference, the interaction between sensory condition and age group arose because interference was greater in unimodal compared with cross-modal conditions but only in older adults (*p* < .001) and not young adults (*p* = .79) or children (*p* = .14).

Quadratic trends did not reach significance for stimulus- or response-interference under unimodal (stimulus-interference: *F*(1, 102) = 5.32, *p* = .02 η_*p*_^2^ = .05, response-interference: *F*(1, 102) = 1.377, *p* = .24, η_*p*_^2^ = .01) or cross-modal (stimulus-interference: *F*(1, 102) = .02, *p* = .88, η_*p*_^2^ < .001, response-interference *F*(1, 102) = .001, *p* = .98, η_*p*_^2^ < .001) conditions.

##### Accuracy

The effects of sensory condition (*F*(1, 102) = 4.29, *p* = .04, η_*p*_^2^ = .04), interference type (*F*(1, 102) = 5.27, *p* = .02 η_*p*_^2^ = .05) and age group (*F*(2, 102) = 5.27, *p* = .47, η_*p*_^2^ = .01) all failed to reach significance. The interaction between sensory condition and age failed to meet significance (*F*(2, 102) = 4.22, *p* = .02, η_*p*_^2^ = .07), as did the interaction between interference type and age group (*F*(2, 102) = .38, *p* = .68, η_*p*_^2^ = .01) and the three way interaction between sensory condition, interference type and age group (*F*(2, 102) = 1.55, *p* = .22, η_*p*_^2^ = .03). However, a significant interaction occurred between sensory condition and interference type (*F*(1, 102) = 11.86, *p* < .001, η_*p*_^2^ = .10). Stimulus- and response-interference only significantly differed from one another under unimodal (*p* < .001) but not cross-modal (*p* = .566) conditions.

Quadratic trends for stimulus- and response-interference did not reach significance under unimodal (stimulus-interference: *F*(1, 102) = .7, *p* = .4, η_*p*_^2^ = .008, response-interference: *F*(1, 102) = 3.25, *p* = .07, η_*p*_^2^ = .03, respectively) or cross-modal (stimulus-interference: *F*(1, 102) = .39, *p* = .53, η_*p*_^2^ = .003, response-interference: *F*(1, 102) = 3.6, *p* = .06, η_*p*_^2^ = .03) conditions.

#### Developmental trajectory analyses

**Figure 4 fig4:**
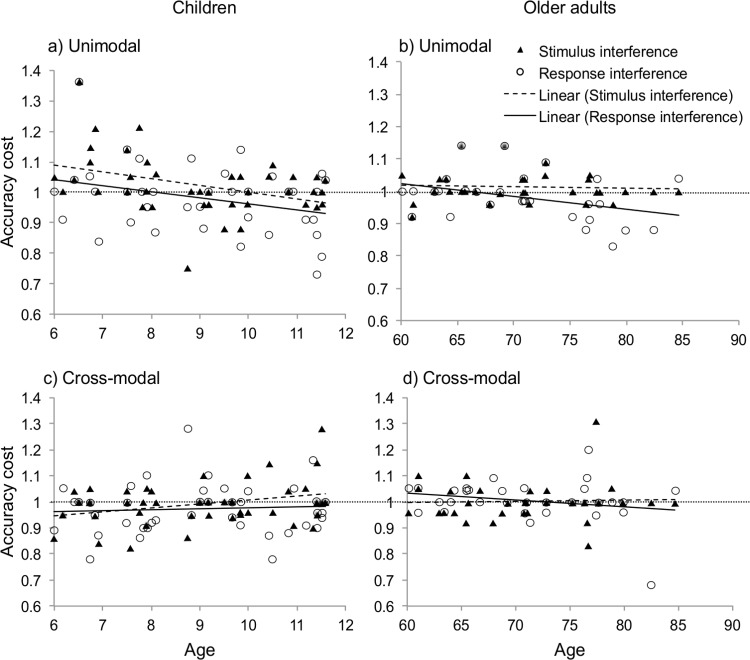
Accuracy ratios for stimulus-interference (SI; triangles and dashed line) and response-interference (RI; circles and continuous line) under unimodal (top) and cross-modal (bottom) conditions. Ratios are shown for children (left) and older adults (right). A significant reduction in accuracy occurred across childhood under unimodal conditions.

Because of the wide age range encompassed within our child (6–11 years) and older adult (60–84 years) samples, two separate 2 (sensory condition) × 2 (interference type) analyses of covariance (ANCOVAs) were performed in which age in decimals was held as a covariate; thus, detecting whether age influenced the pattern of effects observed in these age groups. These analyses were conducted for both response time ratios and accuracy ratios and are shown in Figure 4.[Fig-anchor fig4]

##### Response times

Developmental trajectory analyses on response times yielded no main effects or interactions in adults or children (see Table 1).[Table-anchor tbl1]

##### Accuracy

In children the ANCOVA showed a main effect of sensory condition (*F*(1, 40) = 8.83, *p* = .005, η_*p*_^2^ = .16) that covaried with age (*F*(1, 40) = 8.27, *p* = .006, η_*p*_^2^ = .15). This occurred because unimodal accuracy costs increased (i.e., accuracy was worse) with age while this did not occur under cross-modal conditions. There was no effect of interference (*F*(1, 40) = .77, *p* = .38, η_*p*_^2^ = .02) and this did not covary with age (*F*(1, 40) = .26, *p* = .62, η_*p*_^2^ = .01). There was no interaction between sensory condition and interference type (*F*(1, 40) = 6.09, *p* = .02, η_*p*_^2^ = .12) and this did not covary with age (*F*(1, 40) = 4.97, *p* = .03, η_*p*_^2^ = .1).

It should be noted that the effects of stimulus- and response-interference upon accuracy costs are contradictory. Stimulus-interference would increase accuracy (as the distractor primes to correct response) while response-interference would reduce accuracy (as the distractor primes the incorrect response). Thus, increased accuracy costs (i.e., reduced accuracy) in unimodal conditions across childhood can to be attributed to decreases in stimulus-interference and increases in response-interference with development (Figure 4a; Cragg, 2016).

In older adults the effects of sensory condition and interference type did not reach significance (*F*(1, 30) = .43 *p* = .52, η_*p*_^2^ = .02 and *F*(1, 30) = 2.7 *p* = .11, η_*p*_^2^ = .07, respectively). Neither of these effects were shown to covary with age (*F*(1, 30) = .63, *p* = .43, η_*p*_^2^ = .02 and *F*(1, 30) = 3.34, *p* = .08, η_*p*_^2^ = .09, respectively). There was no interaction between sensory condition and interference type (*F*(1, 30) = .016, *p* = .9, η_*p*_^2^ = .001).

#### Response time distributions

Unimodal response-interference but not cross-modal response-interference increased accuracy costs (see above). Developmental trajectory analyses also suggested increased unimodal, but not cross-modal, accuracy costs across childhood. Together these findings suggest that response-interference may occur under unimodal conditions (and, thus, reduce accuracy) but not cross-modal conditions.

**Figure 5 fig5:**
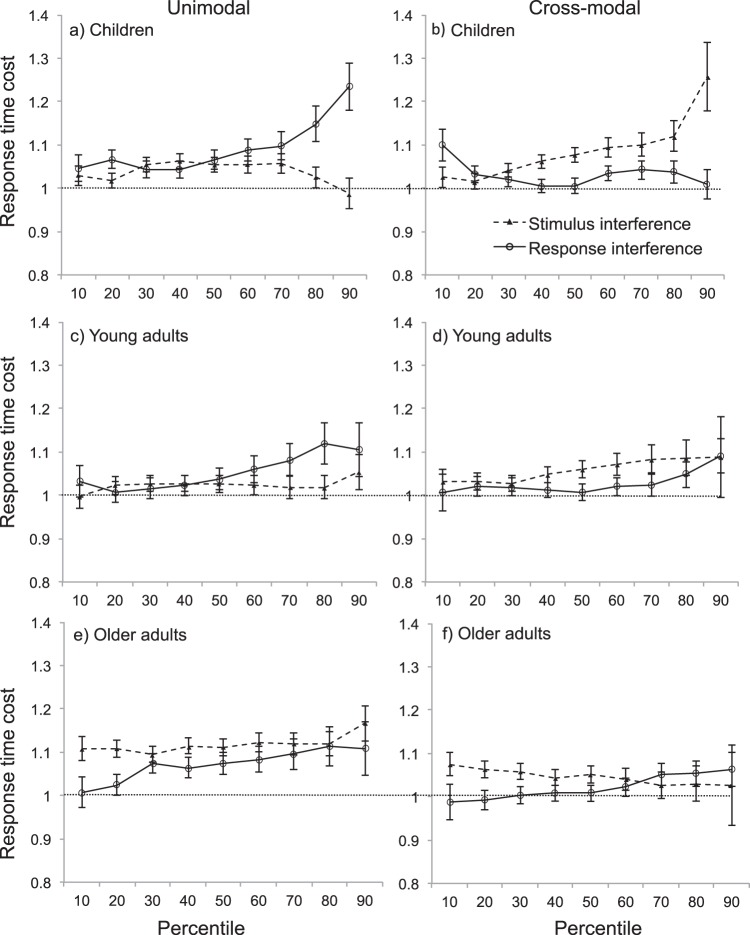
Response time distributions for stimulus- and response-interference under unimodal (left) and cross-modal (right) conditions in children (a/b) young adults (c/d) and older adults (e/f). Each data point shows the mean response times for 10–90th percentiles. Error bars indicate *SE*.

On the other hand, our initial analyses suggested stimulus- and response-interference both slowed response times under unimodal and cross-modal conditions. One possible explanation for contradictory results in response time and accuracy data might be that we find no differences in response times because we collapsed analyses across the response time distribution. Chen et al. (2011) show that stimulus- and response-interference occur at different time-points across the response time distribution. Response-interference occurs at longer latencies, while stimulus-interference appears uniformly distributed across response times. To address this, the 10–90th percentiles for each participant’s response time distributions were identified (see Figure 5). If interference occurs at specific time points in the response time distribution we expected a main effect of percentile. If stimulus- and response-interference occur at different latencies, as predicted by Chen et al. (2011), we expect a percentile by interference type interaction. If unimodal and cross-modal stimulus- and response-interference are different we predict a three-way interaction between percentile, interference type and sensory condition. Finally, if these differences are only observed in some and not all age groups a four-way interaction would be expected.[Fig-anchor fig5]

A 2 (sensory condition) × 2 (Interference type) × 9 (percentile) × 3 (age group) ANOVA showed no main effect of sensory condition (*F*(1, 102) = 4.75, *p* = .03, η_*p*_^2^ = .04), interference type (*F*(1, 102) = .550, *p* = .46, η_*p*_^2^ = .01) or age group (*F*(2, 102) = 2.56, *p* = .08, η_*p*_^2^ = .05). There was a main effect of percentile (Greenhouse-Geisser corrected *F*(2.081, 229.12) = 14.439, *p* < .001, η_*p*_^2^ = .12). There was no two-way interaction between interference type and percentile (Greenhouse-Geisser corrected *F*(2.24, 228.03) = 1.039, *p* = .362, η_*p*_^2^ = .01), and the three way interaction between percentile, interference type and sensory condition did not reach significance (Greenhouse-Geisser corrected *F*(2.25, 229.12) = 2.53, *p* = .08, η_*p*_^2^ = .02). There was, however, a significant four way interaction between percentile, sensory condition, interference type and age (Greenhouse-Geisser corrected *F*(4.49, 229.12) = 4.05, *p* = .002, η_*p*_^2^ = .07).

Post hoc *t* tests showed that in children unimodal response-interference was significantly higher than stimulus-interference at the 90th percentile (*p* = .002). Conversely, under cross-modal conditions, the opposite pattern occurred and stimulus-interference was significantly higher than response-interference at the 90th percentile (*p* = .01). In young adults, and older adults, stimulus- and response-interference did not significantly differ from one another at any point in the response time distribution, under unimodal or cross-modal conditions.

The analysis of response time distribution indicated that in children unimodal and cross-modal interference occurring at longer latencies arose from different types of interference. Under unimodal conditions response-interference was highest at longer latencies while stimulus-interference remained stable across response times. Under cross-modal interference, stimulus-interference peaked at longer latencies, while response-interference remained stable.

### Discussion

Experiment 1 showed unimodal and cross-modal interference differed in multiple ways. First, unimodal interference manifested a U-shape trajectory from childhood, to young adulthood to old age. Cross-modal interference did not manifest a U-shape trajectory and older adults showed substantially more interference under unimodal compared with cross-modal conditions. Second, unimodal but not cross-modal response-interference reduced accuracy. Third, between the ages of 6 and 11 years, accuracy decreased for unimodal, but not cross-modal tasks, although note that this may have resulted from the facilitatory influence of stimulus-interference conditions in early childhood. Finally, in childhood, unimodal and cross-modal interference showed opposing patterns of stimulus- and response-interference across the response time distribution. Under unimodal conditions response-interference peaked at longer response times while stimulus-interference remained stable. Under cross-modal conditions stimulus-interference peaked at longer latencies while response-interference remained stable.

However, “cross-modal” distractions in Experiment 1 were always auditory distractors while participants focused on vision. Thus, results may have occurred because of the distractor being auditory in nature as opposed to cross-modal per se. Evidence suggests a shift in sensory weighting across the life span, such that children prefer auditory information while adults give precedence to visual information (Barnhart, Rivera, & Robinson, 2018; Colavita, 1974; Diaconescu, Alain, & McIntosh, 2011; Hirst et al., 2018; Nava & Pavani, 2013). Furthermore, older adults with mild levels of age-related hearing loss show cortical reorganization, such that auditory cortices are recruited for visual tasks (Campbell & Sharma, 2014). Given shifts in sensory weighting and cortical allocation, it might be expected that children would be more susceptible to distraction from auditory sources while older adults are more susceptible to distraction from visual sources. Indeed, it has been shown that older adults appear able to ignore audition while focusing on vision but not vice versa (Guerreiro, Adam, et al., 2014; Guerreiro, Anguera, et al., 2014; Guerreiro et al., 2010, 2013; Van Gerven & Guerreiro, 2016). Nevertheless, functional magnetic resonance imaging (fMRI) studies have shown age-equivalent suppression of activity in visual cortices during auditory attention (Guerreiro et al., 2015).

Given the existing literature, we would predict that older adults should be able to focus on vision while ignoring audition (as seen in Experiment 1) but should find it more difficult to ignore vision while focusing on audition. Furthermore, based on shifts in sensory dominance, we predict that young children should find auditory distractors more difficult to ignore while focusing on vision than vice versa.

## Experiment 2

Experiment 2 aimed to explore whether the pattern of results seen in Experiment 1 held for two types of cross-modal distraction: focusing on vision while ignoring auditory distractors (as in Experiment 1) and focusing on audition while ignoring visual distractors.

### Method

#### Participants

Sample size calculation was conducted in accordance with Experiment 1, indicating a need for a minimum of 29 participants per age group (87 in total), a criterion that was met by all three samples. Thirty young adults (mean age 25.79 years, range 22–33, 21 female), 53 children (mean age 9.38 years, range 6–11 years, 32 female) and 36 older adults (mean age 71.38 years, range 60–84 years, 21 female) took part. All participants were recruited using the same methods reported in Experiment 1.

One child was excluded because of developmental disorders reported by parents. Five older adults were later excluded, two because of inability to derive an appropriate hearing threshold and three because of the use of hearing aids.

#### Equipment

The equipment used was identical to those detailed in Experiment 1. However, the experimenter used a headphone splitter to check that responses being made in the practice trials were correct. This was necessary as in the new cross-modal condition participants had to respond to a spoken word.

#### Stimuli

Stimuli were identical to those used in Experiment 1 apart from that neutral trials contained no information other than the relevant dimension (i.e., a colored rectangle to be identified or a spoken word to be identified). As in Experiment 1, thresholds were assessed to present all stimuli at 10× (20 dB) above threshold with a maximum visual contrast of 100% and auditory presentation of 65 dB. Auditory stimuli had to be presented at maximum for 27 (of 52) children, 2 (of 30) younger adults and 16 (of 30) older adults included in the final analysis. However, stimuli were still judged to be clearly audible in these participants (*M* = 16.67 dB above threshold range = 6–20 dB above threshold).

#### Procedure

The task was identical to that used in Experiment 1, however, there was no unimodal condition (i.e., ignoring written words while focusing on the color of the rectangle) and this was replaced with a second type of cross-modal condition. The “focus on vision”/“ignore auditory” condition was identical to the cross-modal condition used in Experiment 1. In this condition participants were instructed to sort colored “tickets” (rectangles) into two boxes based on the color of the rectangle while ignoring a spoken word. In the new “focus on audition”/“ignore visual” condition participants were instructed to sort the tickets based on the color-word they heard, while ignoring the actual color of the ticket. An emphasis was made that participants were not allowed to close their eyes and must focus on the ticket at all times.

### Analysis and Results

#### Replication of ignore auditory result

The first goal of Experiment 2 was to replicate the findings from the cross-modal condition of Experiment 1 (i.e., compare the cross-modal condition of Experiment 1 to the ignore auditory condition in Experiment 2). Two 2 (experiment) × 3 (age group) ANOVAs were used to compare general interference ratios in terms of response time and accuracy between the three age groups. Two further 2 (experiment) × 2 (interference type) × 3 (age group) ANOVAs were used to compare stimulus- and response-interference between age groups. We report Bayes factors alongside frequentist statistics to investigate support for the null-hypothesis that Experiment 1 and 2 would not significantly differ from one another with regards to general interference, or stimulus- and response-interference, in each of the three age groups.

Table 2 shows the resulting statistics comparing general interference and stimulus- and response-interference (in terms of response time and accuracy) between experiments. Critically both sets of analyses indicated no significant difference between Experiment 1 and 2 (for all models and effects see online supplementary material S4). Bayesian analysis in all cases were in favor of the null compared with the alternative and this was most convincing when considering stimulus- and response-interference separately. Differences in accuracy between experiments were 5.24 times more likely under the null hypothesis and differences in response times were 3.7 times more likely under the null hypothesis. Thus, we consider the ignore auditory condition of Experiment 2 as a replication of the cross-modal condition in Experiment 1.[Table-anchor tbl2]

#### Auditory versus visual distractors

The main goal of Experiment 2 was to explore whether the findings from Experiment 1 generalized to cross-modal distraction occurring in the opposing modality. If this were the case, we would expect that the U-shape trajectory seen across age groups in unimodal conditions in Experiment 1 would not be seen with either type of cross-modal distractor (older adults would be good at ignoring both auditory and visual distractors). Alternatively, if the findings from Experiment 1 could be explained because of the nature of the cross-modal distractor, we would expect older adults to maintain the ability to ignore auditory distractors while focusing on vision, but not vice versa.

**Figure 6 fig6:**
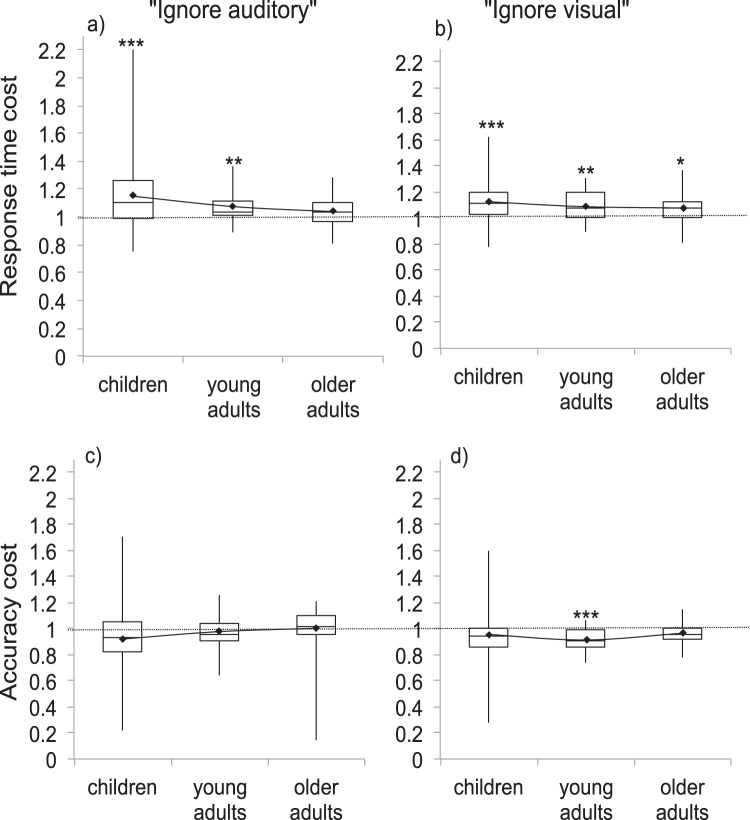
Response time (top) and accuracy (bottom) ratios for general interference in cross-modal conditions with auditory distractors (left) and visual distractors (right) in children, young adults and older adults. Response time, ratios higher than 1 indicate slowing. Accuracy ratios lower than 1 indicate accuracy decrements. Black diamonds indicate means used for analyses. The line connecting means demonstrates the extent of the quadratic (U-shape) trajectory. Asterisks indicate *t* test comparing ratio to 1 (*.05, **.01, ***.001) Bonferroni corrected for six comparisons.

**Figure 7 fig7:**
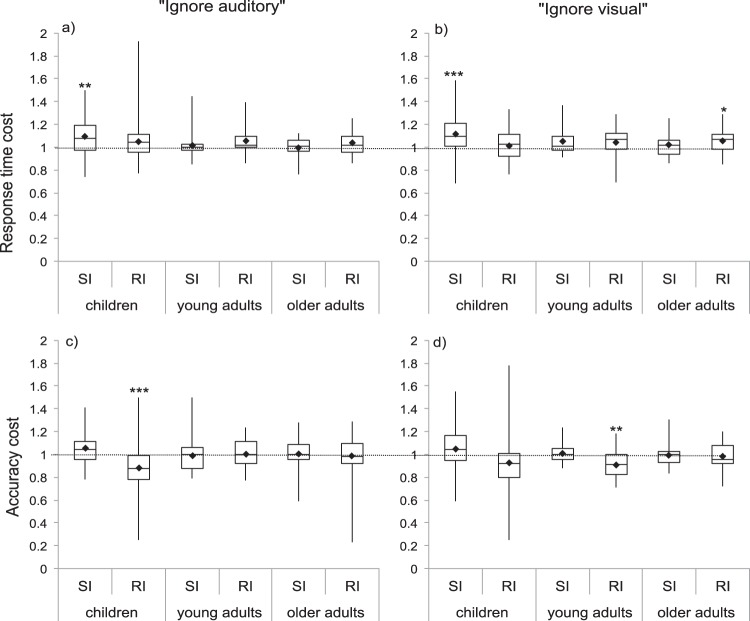
Stimulus- (SI) and response-interference (RI) with auditory (left) and visual (right) cross-modal distractors in terms of response time (top) and accuracy (bottom). Response time ratios greater than 1 indicate slowing. Accuracy ratios less than 1 indicate accuracy decrements. Black diamonds show means used for analyses. Asterisks indicate *t* test comparing ratio to 1 (*.05, **.01, ***.001) Bonferroni corrected for 12 comparisons.

As in Experiment 1, we first examined general interference with both auditory and visual cross-modal distractors in each of the age groups (see Figure 6). We then followed this by separating stimulus- and response-interference (see Figure 7).[Fig-anchor fig6][Fig-anchor fig7]

#### General interference

##### Response times

A 2 (distractor type: auditory, visual) × 3 (age group: children, young adults, older adults) ANOVA showed a main effect of age group (*F*(2, 109) = 4.5, *p* = .01, η_*p*_^2^ = .08) Children showed more interference than older adults, but this did not reach our conservative criterion for statistical significance (*p* = .02). There was no significant difference between general interference in children and young adults (*p* = .12) or younger and older adults (*p* = 1). There was no difference between the two distractor types (*F*(1, 109) = .18, *p* = .67, η_*p*_^2^ = .002). There was no significant interaction between distractor type and age group (*F*(2, 109) = .85, *p* = .43, η_*p*_^2^ = .02).

##### Accuracy

A 2 × 3 ANOVA showed no main effect of distractor type (*F*(1, 109) = .67, *p* = .41, η_*p*_^2^ = .01) or age group (*F*(2, 109) = 1.48, *p* = .23, η_*p*_^2^ = .03). There was no interaction between distractor type and age group (*F*(2, 109) = 1.18, *p* = .31, η_*p*_^2^ = .02).

#### Stimulus and response-interference

##### Response times

A 2 (distractor type) × 2 (interference type) × 3 (age group) ANOVA showed a main effect of age group (*F*(2, 109) = 5.056, *p* = .01, η_*p*_^2^ = .09). Children showed significantly more interference (slowing) than older adults (*p* = .01) but not young adults (*p* = .11). Young adults did not significantly differ from older adults (*p* = 1). There was no effect of interference type (*F*(1, 109) = .074, *p* = .79, η_*p*_^2^ = .001) but age interacted with the effect of interference type (*F*(2, 109) = 5.024, *p* = .01, η_*p*_^2^ = .08). Children showed more stimulus-interference compared with younger and older adults; however, only the comparison between children and older adults reached significance (*p* = .02 and *p* = .001, respectively). Children did not differ from younger or older adults with regards to response-interference (*p* = 1 for both comparisons). Stimulus-interference caused significantly more slowing compared with response-interference in children (*p* = .003) but this difference was not significant in young adults (*p* = .62) or older adults (*p* = .18). There was no effect of distractor type (*F*(1, 109) = .325, *p* = .57, η_*p*_^2^ = .003) and no three-way interaction (*F*(2, 109) = 0.24, *p* = .79, η_*p*_^2^ = .004).

##### Accuracy

A 2 × 2 × 3 ANOVA showed a significant effect of interference type (*F*(1, 109) = 14.12, *p* < .001, η_*p*_^2^ = .11) that interacted significantly with age group (*F*(2, 109) = 5.95, *p* = .004, η_*p*_^2^ = .09). Accuracy costs were larger for response-interference versus stimulus-interference (this finding did not occur under Experiment 1) and this difference only reached significance in children (*p* < .001) and not young adults (*p* = .12) or older adults (*p* = .86). Notably, this may also have occurred because stimulus-interference facilitated correct responses in children (producing mean ratios higher than 1), resulting in differences in accuracy between stimulus- and response-interference.

The main effects of age group and distracter type did not reach significance (*F*(2, 109) = .64, *p* = .53, η_*p*_^2^ = .01; *F*(1, 109) = .19, *p* = .67, η_*p*_^2^ = .002). There was no interaction between distractor type and age group (*F*(1, 109) = 1.35, *p* = .26, η_*p*_^2^ = .02), no interaction between distractor type and interference type (*F*(1, 109) = .18, *p* = .67, η_*p*_^2^ = .002) and no three way interaction between distractor type, interference type and age group (*F*(2, 109) = 2.12, *p* = .13, η_*p*_^2^ = .04).

### Discussion

The results from Experiment 2 suggested that neither type of cross-modal distraction produced the U-shape trajectory seen in the unimodal condition in Experiment 1. Rather, older adults appeared able to ignore both types of cross-modal distraction.

Notably, in Experiment 1 we found that only unimodal and not cross-modal conditions produced accuracy costs (i.e., accuracy decrements) because of response-interference (there was a main effect of interference type that interacted with sensory condition). In Experiment 2, however, we did find a main effect of interference type for cross-modal distraction. Response-interference produced accuracy costs with both visual and auditory distractors. This difference, however, arose only from children and may, in part, be inflated by facilitation on stimulus-interference trials in children. Increased stimulus-interference in children was evidenced in response times in Experiment 2, in which children were significantly slower than young adults and older adults but this was a result of stimulus-interference.

## General Discussion

To our knowledge, this is the first study to compare unimodal and cross-modal Stroop interference across childhood and old age while also considering sensory differences based on individual thresholds, and the first study to separate stimulus- and response-interference within a cross-modal paradigm. We investigated (a) whether unimodal and cross-modal interference follow similar patterns of development and deterioration across the life span; (b) if stimulus- and response-interference contribute to cross-modal, as well as unimodal, interference; and (c) whether the relative contribution of stimulus- and response-interference under unimodal and cross-modal conditions changes across the life span. Overall the findings indicated that different mechanisms underpin unimodal and cross-modal interference-control and that these mechanisms are differentially susceptible to developmental maturation and aging. We begin this section by addressing each research question in turn before focusing in detail on interference-control in older adults and children.

We found that unimodal and cross-modal interference do not follow similar patterns of development and deterioration. Experiment 1 showed that unimodal interference was highest in children and older adults as compared with younger adults, producing a U-shape trajectory. Children also struggled to ignore auditory distractors while focusing on vision. However, older adults maintained the ability to ignore audition while focusing on vision. This finding was replicated in Experiment 2 where we compared two different cross-modal conditions. As such unimodal and cross-modal interference do not appear to follow the same patterns of development and deterioration across the life span.

Stimulus- and response-interference were found to contribute differentially to cross-modal and unimodal interference. Experiment 1 showed that unimodal interference arose from both stimulus and response-interference while cross-modal interference arose mainly from stimulus-interference. Under unimodal conditions, response times were slowed by the presence of conflicting information mapped to the same response. Participants were further slowed, and made errors, if the conflicting information was mapped to a different response. However, cross-modal response-conflict was not sufficient to produce accuracy decrements. This was also the case for adults, but not children, in Experiment 2. This suggests cross-modal interference arises mainly from stimulus-interference while unimodal interference takes effect at both the stimulus and response processing levels.

The relative contribution of stimulus- and response-interference under unimodal and cross-modal conditions was found to change across the life span. It has been proposed that children show more stimulus- than response-interference compared with adults (Cragg, 2016). Our findings support this, but also suggest the contribution of stimulus- and response-interference in childhood may differ between unimodal and cross-modal conditions. In Experiment 1 younger children were more accurate on stimulus-interference trials (showing facilitatory effects). This suggests more stimulus-interference in childhood. However, when comparing the pattern of stimulus and response-interference across the response time distribution under unimodal and cross-modal conditions, children showed different patterns under unimodal and cross-modal conditions. Under unimodal conditions response-interference peaked at the longest response time latencies, while stimulus-interference remained constant (Chen et al., 2011). However, the opposite pattern was seen under cross-modal conditions. Experiment 2 also suggested children were more susceptible to stimulus-interference, as children, but not adults, were significantly slowed by stimulus-interference. Furthermore, accuracy in children was significantly lower on response-interference compared with stimulus-interference conditions, which, as in Experiment 1, may have partly resulted from facilitation of accuracy on stimulus-interference trials. In Experiment 2 cross-modal distractors only reduced accuracy in children. Thus, children appear to process cross-modal distraction differently from younger and older adults and this may in part be because of cross-modal interference occurring at different levels (i.e., also at the response selection levels) of processing in childhood.

### Maintained Cross-Modal Interference-Control in Aging

The findings from Experiments 1 and 2 provided substantial support for the hypothesis that cross-modal interference is less susceptible to age-related decline (Guerreiro et al., 2010). Surprisingly, we found this was the case for both visual and auditory cross-modal distractions. This result is in line with fMRI data suggesting equivalent down-regulation of visual and auditory processing in older and younger adults during cross-modal attention (Guerreiro et al., 2015). However, this finding contradicts findings showing older adults may suppress audition while focusing on vision but not vice versa (Guerreiro, Adam, et al., 2014; Guerreiro, Anguera, et al., 2014; Van Gerven & Guerreiro, 2016). One explanation of differing findings proposed by Guerreiro et al. (2015) is that asymmetrical effects (i.e., an ability to ignore audition while focusing on vision but not vice versa) are seen in tasks in which auditory and visual information are presented concurrently, but symmetrical effects (i.e., an ability to ignore both visual and auditory cross-modal distraction) might occur when information is presented sequentially. In contrast to this, we find symmetrical effects in a task where stimuli were presented simultaneously. Two further explanations may account for the maintained suppression of visual and auditory distractors seen in our study. First, the perceptual load of our task may have been higher, permitting fewer cognitive resources for distractibility (Matusz et al., 2015). Second, it is arguable that our ignore visual condition in Experiment 2 was easier than our ignore auditory condition (see Limitations and Future Directions section).

It is possible that the demands of our task and the number of stimuli presented (color rectangle, written words/visual babble, spoken words/auditory babble, and Brown noise) may have required more cognitive resources compared with previous literature (Guerreiro, Adam, et al., 2014; Guerreiro, Anguera, et al., 2014). Indeed, we presented background noise throughout, and it has been shown that older adults require more cognitive resources to decipher speech in noise (Getzmann, Wascher, & Falkenstein, 2015) and systematic reviews support a link between speech in noise comprehension and cognitive ability (Dryden, Allen, Henshaw, & Heinrich, 2017). Accumulating evidence also suggests increased perceptual effort of general speech processing in aging (Gagné, Besser, & Lemke, 2017). For example, using a dual task paradigm, Tun, Mccoy, and Wingfield (2009) found that, even when words were presented at suprathreshold intensities, older adults showed poorer performance on a secondary task (tracking a mouse on a screen) when recalling auditory information. Combined, the effects of increased perceptual load and increased perceptual effort of listening in older adults may have left fewer cognitive resources to be allocated to visual information; thus, reducing cross-modal visual distraction (Lavie, 1995). Indeed it has been shown that limited cognitive resources may sometimes shield younger children from cross-modal distraction (Matusz et al., 2015). Future research should aim to investigate whether perceptual load, listening effort and dual task performance may predict the extent to which older adults are distracted in cross-modal environments.

Top-down modulation of relevant and irrelevant sensory cortices has been proposed as one mechanism underlying cross-modal interference control. For example, it has been observed that focus on vision results in the suppression of activity in auditory cortex (Ghatan, Hsieh, Petersson, Stone-Elander, & Ingvar, 1998; Johnson & Zatorre, 2005; Kawashima, O’Sullivan, & Roland, 1995; Mozolic et al., 2008; Weissman et al., 2004). Neuroimaging studies investigating this process in aging appear mixed. Some findings show older adults manifest less down-regulation of auditory cortices when processing visual information (Hugenschmidt, Mozolic, Tan, Kraft, & Laurienti, 2009) and some suggest age-equivalent suppression of visual and auditory cortices in cross-modal attention (Guerreiro et al., 2015) while others suggest intact suppression of auditory but not visual processing in cross-modal attention (Guerreiro, Anguera, et al., 2014). Other neuroimaging studies also suggest different, compensatory strategies may be used in older adults when ignoring irrelevant information (Allen & Payne, 2012). It is possible that this may also be the case for cross-modal control. Peiffer et al. (2009) found that older adults inhibit distinctly different regions of occipital cortex when ignoring visual information compared with young adults. Similarly, Diaconescu, Hasher, and McIntosh (2013) found posterior parietal and medial frontal activity in older adults was increased in older adults relative to younger adults when presented with cross-modal stimuli, and found that this activity was related to faster detection of cross-modal stimuli. Thus, the neural mechanisms used to support cross-model interference control in aging may undergo reorganization, which may help support the normal behavioral performance seen in our study.

### Increased Stimulus-Interference and Differential Processing of Cross-Modal Distraction in Childhood

Our findings support the claim that children experience more stimulus-interference compared with adults (Cragg, 2016). In Experiment 1 these effects are seen in our developmental trajectory analysis, in which younger children counterintuitively manifested higher accuracy under unimodal conditions, which decreased with age. As shown in Figure 4 this likely arose because of the combined effects of increasing response-interference and decreasing stimulus-interference across childhood. Increased response-interference in later childhood resulted in accuracy decrements, as the incongruent distractor primed an incorrect response. Conversely, increased stimulus-interference in early childhood resulted in increased accuracy, as the incongruent distractor primed/facilitated the correct response. Curiously, this effect was not seen under cross-modal conditions. In Experiment 2, both types of cross-modal distractor slowed response times in children, but this appeared attributable to stimulus-interference. Together, these findings suggest that younger children experience more stimulus-interference. Furthermore, the balance of stimulus- versus response-interference in unimodal distraction changes from early to late childhood, while this does not appear to be the case for cross-modal distraction.

Across experiments, children appeared to process cross-modal distraction differently from adults and, as a result, were more susceptible to cross-modal distraction. In Experiment 1 unimodal stimulus- and response-interference followed similar response time distributions to those reported in adults, with response-interference peaking at longer response latencies (Chen et al., 2011). However, children showed the opposite pattern under cross-modal conditions, with stimulus-interference peaking at longer response latencies. This might explain why children, but not adults, were susceptible to cross-modal distractions in Experiment 2. It has been proposed that peripheral mechanisms may filter out cross-modal distraction at earlier processing stages (Guerreiro et al., 2010), and this is consistent with the current findings that cross-modal interference arises at stimulus-encoding stages. However, if cross-modal stimulus-interference peaks later in time in children, this suggests cross-modal distractors are more difficult to suppress at peripheral stages in childhood. Furthermore, given that cross-modal distractors reduced children’s accuracy in Experiment 2, it is possible that cross-modal interference also occurs at later response selection stages in childhood. However, given that this was not clearly evidenced in Experiment 1 these conclusions remain speculative, and warrant further investigation. Nevertheless, our findings suggest differential processing of unimodal and cross-modal distractors in childhood, and suggest children are more susceptible to cross-modal distraction. Support for this is seen in the differences in response time distribution between unimodal and cross-modal distractors in children (Experiment 1), different developmental trajectories for unimodal and cross-modal distraction (Experiment 1), and heightened cross-modal interference (increased slowing and reduced accuracy) in childhood (Experiment 2).

### Limitations and Future Directions

The current study provides important findings in an underrepresented area of literature. However, considering some limitations may guide future research. First, within this study we focus on age groups in which multisensory and interference control processes are known to be immature, below 11 years (Hirst, Stacey, et al., 2018; Noel et al., 2016), and susceptible to age-related decline, above 64 years (Comalli et al., 1962; Noel et al., 2016). However, it has been shown that multisensory integration processes continue to mature across adolescents until around 17 years of age (Innes-Brown et al., 2011; Noel et al., 2016) and temporal binding windows progressively increase between the ages of 50 and 64 (Noel et al., 2016). Furthermore, asymmetries in unimodal stimulus- and response-interference have been reported between adolescents (who manifest more response-interference) and middle-aged adults (who show more stimulus-interference; Killikelly & Szűcs, 2013). Investigating these age groups may, thus, provide insight into the complete life span trajectories of unimodal and cross-modal interference.

Second future research should aim to optimize comparisons between sensory conditions. In our study we implemented a variant of the established De Houwer (2003) paradigm that has been utilized in developmental (Killikelly & Szűcs, 2010) and aging (Killikelly & Szűcs, 2013) contexts to separate stimulus and response-interference. Using our variant we were able to compare unimodal and cross-modal conditions in which visual information was relevant (Experiment 1) and compare cross-modal conditions in which visual and auditory information was relevant (Experiment 2). Furthermore, through using a color patch version of the Stroop task we aimed to prevent confounding unimodal and cross-modal conditions with integrated versus separate Stroop tasks (Macleod, 1998). Nevertheless, this design had some limitations. First, there was no unimodal auditory condition. We recognize that “fully crossed” paradigms are underrepresented in the literature (Guerreiro et al., 2010; Van Gerven & Guerreiro, 2016) and future research should implement such designs to enable full comparison between unimodal and cross-modal interference control. Second, in Experiment 2, the relevant visual dimension was a color, while the relevant auditory dimension was a word. Stroop interference has been attributed to imbalanced ease of access to color versus word information, whereby color naming is more difficult than word reading (Melara & Algom, 2003; Roelofs, 2005). It is, therefore, possible that the focus auditory condition was easier than the focus visual condition. Given this, we cannot exclude the possibility that older adults were able to perform this task because of the ease of access to spoken word information. This might be one explanation as to why we find older adults are able to ignore auditory information while focusing on vision, while other research has found older adults can ignore audition while focusing on vision but *not* vice versa (Van Gerven & Guerreiro, 2016). However, we would still expect focusing on audition while ignoring vision to be difficult in older adults, given age-related increases in listening effort (Tun et al., 2009), reduced dual task performance (Gagné et al., 2017; Verhaeghen, Steitz, Sliwinski, & Cerella, 2003), reduced speech in noise comprehension (Getzmann et al., 2015), and shifts in sensory dominance in favor of vision (Diaconescu et al., 2013). Nevertheless, future research should be able to decipher whether ease of word access may account for maintained ability in aging by equating the type of visual and auditory stimuli in cross-modal tasks.

A final aspect of our design that should be emphasized is that we used a color patch Stroop as opposed to a standard color-word Stroop paradigm. As noted previously, this design was used so that both unimodal and cross-modal conditions could be considered “separated” Stroop tasks (Macleod, 1998). However, it is likely that this design may account for the smaller Stroop effects seen in our study (many interference scores did not significantly differ from baseline, as shown in Figures 2, 3, 6, and 7). Use of integrated paradigms may provide a means of producing larger effects to compare between unimodal and cross-modal paradigms.

## Conclusions

The findings from this study suggest that the ability to ignore distraction within and across senses undergo different life span trajectories. Our findings form empirical support for the theory that cross-modal interference is less susceptible to age-related decline (Guerreiro et al., 2010; Van Gerven & Guerreiro, 2016) but extend this to show that older adults may be able to ignore audition while focusing on vision and vice versa. Conversely, children appear more susceptible to both unimodal and cross-modal distractions compared with adults. This might be because children process cross-modal distractions differently; however, this warrants further investigation. If you are still imagining you are reading this article in your favorite café, the findings of the present study mean that if you are an older adult you might be able to ignore people chatting at the next table better than you thought.

## Supplementary Material

10.1037/xhp0000608.supp

## Figures and Tables

**Table 1 tbl1:** Results From 2 (Sensory Condition: Unimodal, Cross-Modal) × 2 (Interference Type: Stimulus-, Response-Interference) ANCOVA on Response Times in Children and Older Adults

	Children				Older adults			
Effect	*df*	*F*	*p*	η_*p*_^2^	*df*	*F*	*p*	η_*p*_^2^
Sensory Condition	1	2.13	.15	.05	1	.214	.65	.01
Sensory Condition × Age	1	1.57	.22	.04	1	.915	.35	.03
Interference type	1	.03	.87	.00	1	.58	.58	.01
Interference Type × Age	1	.00	.96	.00	1	.5	.49	.02
Sensory Condition × Interference Type	1	.61	.44	.02	1	.44	.51	.02
Sensory Condition × Interference Type × Age	1	1.05	.31	.03	1	.475	.5	.02
*Note*. ANCOVA = analysis of covariance. No significant effects were found. The residual degrees of freedom for all comparisons with children and older adults were 40 and 30 respectively.								

**Table 2 tbl2:** ANOVA and Bayesian Statistics for Comparison of Effects Between Experiment 1 and 2 for Accuracy and Response Times (RT)

General interference										
	Accuracy					RT				
Models	BF_M_	BF_01_	*F*	*p*	η_*p*_^2^	BF_M_	BF_01_	*F*	*p*	η_*p*_^2^
General interference										
Null model	2.89	1.00				1.25	1.00			
Experiment	.76	**2.63**	1.35	.25	<.01	.73	**1.54**	1.51	.22	<.01
Stimulus- and response-interference										
Null model	.001	1.00				11.07	1.00			
Experiment	2.9e–4	**5.24**	1.19	.28	<.01	2.35	**3.7**	1.5	.22	<.01
*Note*. ANOVA = analysis of variance. BF_M_ = change from before posterior model odds (for corresponding posterior and prior odds see supplementary Tables in S4). BF_01_ = Bayes factor for each model against the alternative (favor for the null). Bayes factors highlighted in bold indicate no clear effect of experiment, showing stronger support for the null.										

## Footnotes

1 All experimental materials and data are available via https://osf.io/vmdz9/.

2 We also outline the implications of results in which *p* ≤ .05 in the online supplementary material section S4.2.
