# Supplementary material for: Cross-Modal Interference-Control Is Reduced in Childhood but Maintained in Aging: A Cohort Study of Stimulus- and Response-Interference in Cross-Modal and Unimodal Stroop Tasks
Source: J Exp Psychol Hum Percept Perform. 2019 Apr 4;45(5):553–72. doi: 10.1037/xhp0000608 (PMC6484713; doi:10.1037/xhp0000608)
Supplement: Supplementary file 1 [file Cross_Modal_Interference_JEPHPP_Supplementary_xhp0000608.docx]

# Supplementary Material

**Manuscript: Cross-modal interference control is limited in childhood but maintained in aging: A lifespan study of stimulus and response interference in cross-modal and** **unimodal Stroop tasks**

Authors: Rebecca, J. Hirst^[[1]](#footnote-1)^*, Ella C. Kicks^[[2]](#footnote-2)^, Harriet, A. Allen1 & Lucy Cragg1

**Content**

S1: Protocol for deriving auditory and visual thresholds in adults and children (in experiment 1 and 2).

S2: Analyses of auditory and visual threshold data (for experiment 1 and 2).

S3: Analyses of raw (i.e. non-ratio) accuracy and response time data for all four congruency conditions (neutral, congruent, stimulus-incongruent and response-incongruent; for experiment 1 and 2).

S4: Additional tables:

S4.1 Full Bayesian statistics for comparison of experiment 1 and 2.

S4.2 Consideration of effects in main manuscript in which *p* <= .05

# S1. Matching Auditory and Visual Stimulus Discriminability

To control for the detectability of auditory and visual distracting words in the Stroop task, thresholds for reading words, on each colour background, and hearing spoken words were measured. In Experiment 1, a staircase procedure was used to derive thresholds in adults and a method of adjustment was used with children to avoid prolonged testing. In Experiment 2 the same method of adjustment was used across all age groups.

## **S1.1 Experiment 1 Threshold Protocol**

### **S1.1.1 Adults**

The protocol implemented in Experiment 1 with adult participants is illustrated in Figure S1. Participants identified the colour-word they saw/heard under visual and auditory conditions respectively by pressing one of four buttons. Two colour-words were mapped to two buttons on the right hand side (press the K key for “RED” and the L key for “GREEN”) and two to the left hand side (press the A key for ”BLUE” and the S key for “YELLOW” on a QWERTY keyboard). The side to which colour-words were mapped was counterbalanced across participants and remained the same across threshold and Stroop tasks in each participant.

A 1-up-3-down staircase converged upon the level at which participants correctly identified words on 79% of trials. Thresholds for each staircase were taken as the average of the final 6 out of 8 reversals and final thresholds were taken as the average of the three derived.

#### **S1.1.1 .1 Visual staircase**

Participants were presented with a coloured rectangle overlaid by a randomly selected written colour word (“RED”, “GREEN”,”BLUE”, or “YELLOW”) for 482ms alongside a randomly selected sample of auditory babble and Brown noise both set to 60dB. Participants were instructed to wait until a question mark appeared following stimulus offset to identify the written word. The contrast of the word adjusted in a 1-up 3-down staircase (Step size (% opacity) =[11.76, 7.84, 3.92, 3.92, 1.96, 0.39, 0.10, 0.04,], start value= 39.21% opacity) until 8 reversals had been reached. Participants completed 12 visual staircases each (three for words on each coloured rectangle) in a random order. Final thresholds were taken as the average of the final 6 reversal values. The colour of the rectangle presented remained constant throughout each block to reduce reverse Stroop effects (i.e. incorrectly responding to the colour of the rectangle rather than the written word).

#### **S1.1.1 .2 Auditory staircase**

**Figure S1.** Adult protocol for isolating visual (a) and auditory (b) thresholds for written and spoken words presented with different colour rectangles. Under visual conditions participants were presented with a written word on a coloured rectangle alongside 60dB auditory babble and Brown noise. Participants were asked to wait until the question mark to identify what word they saw using four possible response options. Under auditory conditions participants were presented with a coloured rectangle containing written babble alongside a spoken colour-word and 60dB Brown noise. Participants were asked to wait until the question mark to identify what word they heard. The intensity (contrast or volume) of the word decreased or increased depending on the participants responses. Participants completed 12 staircases (three for written words on and three for spoken words presented with each coloured rectangle; red, green, blue and yellow).


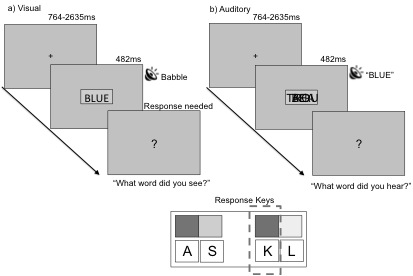


A randomly selected spoken colour word (“RED”, ”GREEN”, ”BLUE”, or “YELLOW”) embedded in 60dB Brown noise was presented alongside a coloured rectangle presented for 482ms overlaid by a randomly selected sample of visual babble. Participants were instructed to wait until a question mark appeared following stimulus offset to identify the *spoken* word. The volume of the spoken word adjusted in a 1-up 3-down staircase until 8 reversals had been reached (Step sizes (dB) = [20, 15, 10, 5, 3, 2, 1, 1], auditory start value= 60dB). Adults completed 12 auditory staircases each (three for spoken words with each coloured rectangle) in a random order. Final thresholds were taken as the average of the final 6 reversal values. The colour of the rectangle presented remained constant throughout each block to reduce reverse Stroop effects (i.e. incorrectly responding to the colour of the rectangle rather than the spoken word).

### **S1.1.2 Children**

Children’s thresholds were isolated via a method-of-adjustment (MOA) illustrated in Figure S2. Children completed 4 visual MOA’s (one MOA for written words on each coloured rectangle) in a random order followed by one MOA for spoken words with a randomly selected coloured rectangle. The reason for utilising one auditory MOA was two-fold. Firstly the public engagement event we tested at placed time constraints upon the amount of time allowed for testing. Secondly, there were no clear differences between the auditory thresholds derived with each coloured rectangle in the adults (see analysis of threshold data supplementary S2).

#### **S1.1.2.1 Visual method-of-adjustment**

A written word on a coloured rectangle was presented alongside a randomly selected sample of auditory babble and Brown noise both set to 60db. Within each block the colour of the rectangle remained the same. The starting contrast of the written word varied depending on its coloured background; yellow= 3.92% opacity, green= 3.92% opacity, red= 5.88% opacity, blue= 7.84% opacity. These starting values were judged based on adult data showing written words were easier to identify in higher contrast conditions (i.e. written words on yellow and green) compared with lower contrast conditions (i.e. written words on blue and red) – see section S2. The experimenter then decreased the contrast of the written word (using 0.04% opacity steps). Each time the experimenter decreased the contrast of the word a new, randomly selected colour word and sample of auditory babble was presented. Children then told the experimenter when they could “only just read the word”.

Five test stimuli were then presented in which participants read aloud a written word set to the reported threshold on the coloured rectangle that had just been presented. A minimum of 4/5 (80%) correct answers was required to move onto the next coloured rectangle, otherwise thresholds for words on that colour were re-measured.

#### **S1.1.2.2 Auditory method-of-adjustment**

Spoken colour-words were presented embedded in 60dB Brown noise. Spoken words were accompanied with a visually presented, randomly selected, coloured rectangle overlaid with a randomly selected patch of visual babble. The spoken word was presented with a starting value of 65dB. The experimenter then decreased the volume of the spoken word (using 1dB steps). Each time the experimenter decreased the volume of the spoken word a new, randomly selected spoken colour-word and sample of visual babble was presented (the colour of the rectangle remained the same throughout to prevent reverse Stroop effects). Children then reported when they could “only just hear the word”.

Five test stimuli were then presented in which a coloured rectangle and a spoken colour-word (set to the reported threshold) were presented. Participants identified the word they heard by repeating it out loud. Four out of five (80%) correct answers were required otherwise the auditory threshold was re-measured.

## **S1.2 Experiment 2 Threshold Protocol**

In Experiment 2 no written words were presented in the Stroop task (participants identified the colour of a rectangle whilst ignoring a spoken word or vice versa). Therefore, only one, auditory, threshold was derived using a single MOA in both adults and children. The method for deriving auditory thresholds in Experiment 2 was identical to auditory MOA procedure described for children in Experiment 1 (and shown in figure S2 b).

**Figure S2.** Method of adjustment protocol used with children in Experiment 1 and all ages in Experiment 2. In the “adjustment phase” the experimenter adjusted the contrast or volume of the word until participants reported they could “only just read/hear the word”. In the “test phase” participants completed 5 test trials in which the word was presented at threshold. A minimum of 80% correct was required otherwise the protocol was repeated to derive a new threshold. In Experiment 1 children completed 5 runs altogether (one for written words on red, green, blue and yellow rectangles and another for spoken words presented with a randomly selected coloured rectangle). In Experiment 2 only the auditory MOA was required.


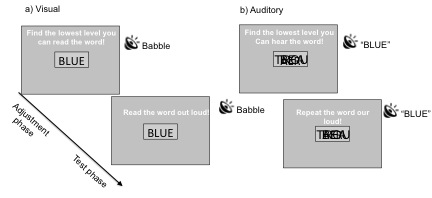


# S2. Analysis of threshold data for Experiment 1 and 2

## **S2.1 Experiment 1**

Mean visual and auditory thresholds for identifying words with different coloured backgrounds are shown in Table S1. To justify methods used with children (i.e. the selected starting values in visual MOA and the use of a single auditory MOA) we first provide analyses of younger and older adults (showing lower visual thresholds on some colour backgrounds compared with others and showing colour background had no effect on auditory threshold). We then follow this with a full analysis comparing thresholds across all age groups.

### **S2.1.1 Visual thresholds**

A 4 (colour background) x 2 (age group; young adults versus older adults) ANOVA comparing visual thresholds in adults showed a main effect of colour background (*F*(3, 183) = 38.38, *p* < .001, $\eta_{p}^{2}$= .39). Thresholds for reading words on yellow (*M =* 4.04% opacity, *SE* = 0.48%) and green (*M =* 3.75% opacity, *SE* = 0.22%) were significantly lower than thresholds for reading words on red (*M =* 6.63%, *SE* = 0.47%) and blue (*M =* 7.63%, *SE* = 0.51%) (*p* < .001 for all comparisons). Thresholds did not significantly differ between green and yellow backgrounds (*p* = 1) or blue and red backgrounds (*p* = .27). This justified our use of lower starting values for yellow and green compared with red and blue for the MOA in children.

A significant interaction was also seen between colour background and age group (*F*(3, 183) = 7.73, *p* < .001, $\eta_{p}^{2}$= .07) this occurred because although there was a main effect of age group (*F*(1, 61) = 7.91, *p* = .007, $\eta_{p}^{2}$= .12), in which thresholds were higher for older adults, this was significant with green (*p* = .008), red (*p* = .004) and blue (*p* < .001) backgrounds but not yellow backgrounds (*p* = .93) in which thresholds were persistently low across younger and older adults.

A 4 (colour background) x 3 (age group) ANOVA comparing visual thresholds across all age groups showed a main effect of age group (*F*(2, 102) = 16.48, *p* < .001, $\eta_{p}^{2}$= .24), a main effect of colour background (*F*(2.78, 283.1) = 67.08, *p* < .001, $\eta_{p}^{2}$= .34) and an interaction between age group and colour background (*F*(5.55, 283.1) = 9.011, *p* < .001,$\eta_{p}^{2}$= .1). The main effect of age occurred as older adults’ visual thresholds were significantly higher than children (*p* < .001) and young adults (*p* = .002) however children and young adults did not differ (*p* = .18). The main effect of colour background occurred as thresholds for detecting words written on green and yellow were significantly lower than thresholds for detecting words on blue and red (*p* < .001 for all comparisons). However thresholds for detecting words on yellow and green did not significantly differ from each other (*p* = 1), as did thresholds for blue and red (*p* = .022). Simple main effects showed the interaction between age group and colour background occurred because the main effect of age group was significant for all colours apart from yellow (*p* < .001 for all compared with *p* = .09), in which thresholds were comparably low across age groups.

### **S2.1.2 Auditory thresholds**

A 4 (colour rectangle; red, green, blue, yellow) x 2 (age group; older adults, younger adults) ANOVA showed a main effect of age group (*F*(1, 61) = 8.75, *p* = .004, $\eta_{p}^{2}$= .13) in which auditory thresholds were higher for older adults. Importantly, there was no significant effect of colour on auditory threshold (*F*(2.65, 161.89) = 1.37, *p* = .257, $\eta_{p}^{2}$= .02) and this did not interact with age group (*F*(2.65, 161.9) = .37, *p* = .75, $\eta_{p}^{2}=$.01). Bayesian analyses showed strong support for the conclusion that auditory threshold did not differ between coloured background as these results were 9.255 times more likely to occur under the null (${BF}_{01}$= 9.255, ${BF}_{10}$= .108)– thus verifying the use of deriving auditory thresholds in the presence of a single coloured background in children.

To compare auditory thresholds across age groups the mean of the auditory thresholds derived in each adult participant was used to compare with the single auditory threshold derived in children. A 3-way ANOVA comparing this single auditory threshold value across age groups showed a significant effect of age group (*F*(2, 102) = 6.73, *p* = .002, $\eta_{p}^{2}=$.12). Older adults auditory thresholds (*M =* 47.56dB, *SE =* .87) were higher than younger adults (*M =* 43.24dB, *SE =* .88, *p* = .002) and

children (*M =* 44.29dB, *SE =* .76, *p* = .017). Auditory threshold did not significantly differ between children and young adults (*p* = 1).

|  | **Red** | **95%CI** | | **Green** | **95%CI** |  | **Blue** | **95%CI** |  | **Yellow** | **95%CI** |  |
| --- | --- | --- | --- | --- | --- | --- | --- | --- | --- | --- | --- | --- |
|  | ***M*** | **Lower** | **Upper** | ***M*** | **Lower** | **Upper** | ***M*** | **Lower** | **Upper** | ***M*** | **Lower** | **Upper** |
| Visual Thresholds (% opacity) | | | | | | | | | | | | |
| Children | 3.98 | 3.07 | 4.89 | 2.86 | 2.44 | 3.30 | 4.78 | 3.78 | 5.79 | 2.71 | 1.79 | 3.62 |
| Young adults | 5.25 | 4.19 | 6.32 | 3.15 | 2.66 | 3.64 | 5.72 | 4.55 | 6.89 | 4.08 | 3.02 | 5.14 |
| Older adults | 8.01 | 6.96 | 9.05 | 4.35 | 3.86 | 4.83 | 9.55 | 8.39 | 10.7 | 3.99 | 2.95 | 5.04 |
| Auditory (dB) | | | | | | | | | | | | |
| Younger adults | 42.97 | 40.76 | 45.18 | 43.9 | 41.44 | 46.37 | 43.32 | 40.76 | 45.18 | 42.77 | 40.73 | 44.82 |
| Older adults | 46.91 | 44.73 | 49.08 | 47.84 | 45.42 | 50.27 | 47.91 | 45.64 | 50.18 | 47.56 | 45.55 | 49.58 |

**Table S1.** Mean (*M*) visual and auditory thresholds and 95% confidence intervals (CI) for reading and hearing words presented with different coloured backgrounds in each age group in Experiment 1.

## **S2.2 Experiment 2**

A 3-way ANOVA comparing auditory thresholds across age groups showed a significant effect of age group (*F*(2, 109) = 32.62, *p* < .001, $\eta_{p}^{2}=$ .37). Older adults’ auditory thresholds (*M =* 47.367dB, *SE =* .65) were higher than younger adults (*M =* 40.1dB, *SE =* .65 p < .001) and children (*M =* 44.75dB, *SE =* .49, *p* = .005). Children’s thresholds were also higher than young adults (*p* < .001).

# S3. Analysis of raw (non-ratio) data

## **S3.1 Experiment 1**

### **S3.1.1 Response times**

Mean response times for each condition in each group are shown in Table S2. These were analysed in a 2 (sensory condition) x 4 (congruency) x 3 (age group) mixed ANOVA.

Analysis revealed a main effect of congruency (*F*(2.65^[[3]](#footnote-3)^, 270.1) = 23.14, *p* < 0.001, $\eta_{p}^{2}=$ .18) and a main effect of age group (*F*(2, 102) = 40.315, *p* < .001, $\eta_{p}^{2}=$ .44). The effect of congruency was not modulated by age group (*F*(5.3, 270.1) = 1.93, *p* = .09, $\eta_{p}^{2}=$ .03), or sensory condition (*F*(2.49, 270.1) = 2.81, *p* = .05,$\eta_{p}^{2}=$ .03) and there was no three way interaction between sensory condition, age group and congruency (*F*(4.98, 254.18) = 2.079, *p* = .069, $\eta_{p}^{2}=$ .04). This suggest that a similar pattern of congruency effects occurred across ages in unimodal and cross-modal tasks. ^[[4]](#footnote-4)^

Response times were significantly faster under congruent (*M =* 703ms, *SE =* 17ms) vs. response-incongruent (*M =* 766ms, *SE =* 19ms) conditions (*p* < .001), congruent vs. stimulus-incongruent (*M =* 748ms, *SE =* 20ms) conditions (*p* < .001) and congruent vs. neutral (*M =* 748ms, *SE =* 19ms) conditions (*p* < .001). Response times did not significantly differ between stimulus- and response-incongruent conditions (*p* = .237). Notably response times did not differ between neutral and stimulus-incongruent conditions (*p* = 1) or neutral and response-incongruent conditions (*p* = .227), suggesting interference occurring on neutral trials. This may have resulted from stimulus novelty (as the word “Brown” was not included during the thresholding procedure) or stimulus-interference itself. Indeed, previous literature has utilised stimuli that are not in the response set to probe stimulus-interference (Milham et al., 2001). For this reason neutral conditions were not used in the calculations of interference ratios noted within our main manuscript.

There was no main effect of sensory condition (*F*(1, 102) = .004, *p* = .95, $\eta_{p}^{2}<$.001), suggesting similar response times between unimodal and cross-modal tasks generally. However the effect of age group was modulated by sensory condition (*F*(2, 102) = 4.589, *p* = .012, $\eta_{p}^{2}=$.08). Children (*M =* 929ms, *SE =* 29ms) were slower than young (*M =* 525ms, *SE =* 34ms *p* < .001) and older (*M =* 770ms, *SE =* 34ms *p* = .001) adults, and older adults were slower than young adults (*p* < .001). The interaction between age group and sensory condition occurred because, under unimodal conditions, children (*M =* 904ms, *SE =* 32ms) and older adults (*M =* 801ms, *SE =* 37ms) were significantly slower than young adults (*M =* 517ms, *SE =* 38ms) (*p* < .001) whilst children and older adults did not differ (*p* = .12). Conversely, under cross-modal conditions, children (*M =* 953ms, *SE*  31ms) were significantly slower than young adults (*M =* 532ms, *SE =* 36ms) and older adults (*M =* 740ms, *SE =* 36ms) (*p* < .001) and older adults were significantly slower than young adults (*p* < .001). This finding coincides with the conclusion in our main manuscript, that children experience more cross-modal distraction compared with adults.

As shown in Table S2 children and older adults manifested larger Stroop effects than younger adults (i.e. they were slower under incongruent vs. congruent conditions compared with young adults). However the interaction between congruency and age group did not reach significance. This is not in line with the age related Stroop effects reported in past unimodal literature (Comalli, Wapner, & Werner, 1962). Notably, the interaction between congruency, sensory condition and age group also did not reach significance, suggesting this was not due to the inclusion of cross-modal conditions.

|  |  | **Unimodal** | **95% CI**  **Lower** | **Upper** | **Cross-modal** | **95%CI**  **Lower** | **Upper** |
| --- | --- | --- | --- | --- | --- | --- | --- |
| Children | C | 851 | 793 | 909 | 905 | 846 | 964 |
|  | SI | 913 | 839 | 987 | 965 | 902 | 1028 |
|  | RI | 944 | 872 | 1016 | 968 | 901 | 1035 |
|  | N | 907 | 839 | 975 | 975 | 908 | 1042 |
| Young adults | C | 499 | 432 | 567 | 513 | 445 | 582 |
|  | SI | 511 | 425 | 597 | 542 | 468 | 615 |
|  | RI | 524 | 440 | 608 | 544 | 466 | 622 |
|  | N | 535 | 456 | 615 | 530 | 452 | 607 |
| Older adults | C | 728 | 662 | 794 | 722 | 654 | 789 |
|  | SI | 808 | 724 | 893 | 749 | 676 | 821 |
|  | RI | 861 | 778 | 944 | 754 | 677 | 831 |
|  | N | 805 | 727 | 883 | 734 | 658 | 811 |

Table S2 RT for each age group under congruent (C) stimulus-incongruent (SI) response-incongruent (RI) and neutral (N) conditions under unimodal and cross-modal sensory conditions for Experiment 1. Confidence intervals (CI) represent the 95% bootstrapped confidence intervals.

### **S3.1.2 Accuracy**

Accuracy scores for each age group under each condition are shown in Table

S3. A 2 (sensory condition) x 4 (congruency) x 3 (age group) mixed ANOVA on the accuracy scores across groups revealed a main effect of congruency (*F*(2.63, 268.14) = 7.48, *p* < .001, $\eta_{p}^{2}=$.08) and a main effect of age group (*F*(2, 102) = 19.48, *p* < .001, $\eta_{p}^{2}=$.28). The main effect of congruency was not modulated by age group (*F*(5.26, 268.14) = 1.026, *p* = .4, $\eta_{p}^{2}=$ .02) suggesting similar congruency effects between groups. There was however a significant interaction between congruency and sensory condition (*F*(3, 306) = 5.16, *p* = .002, $\eta_{p}^{2}=$ .05) suggesting different congruency effects between unimodal and cross-modal conditions. The three way interaction between sensory condition, congruency and age group failed to meet significance (*F*(6, 306) = 2.25, *p* = .04, $\eta_{p}^{2}=$ .04).

Accuracy was significantly lower in response-incongruent (*M =* 90% *SE =* .75) vs. congruent (*M =* 92%, *SE =* .61) (*p* = .001) and response-incongruent vs. stimulus-incongruent (*M =* 92%, *SE =* .66) (*p* = .002) but not response-incongruent vs. neutral (*M =*91%, *SE =* .7) (*p* = .53) conditions. Notably, stimulus-incongruent conditions should not prime errors as irrelevant stimuli are also mapped to the correct responses (thus differences between response incongruent and stimulus incongruent conditions can also result from facilitation on stimulus incongruent trials). Older adults (*M =* 96%, *SE =* 1.03) were significantly more accurate than younger adults (*M =* 90%, *SE =* 1.04, *p* < .001), and children (*M =* 88%, *SE =* .9, *p* < .001). However accuracy between children and adults did not significantly differ (*p* = .298).

The interaction between sensory condition and congruency occurred because the main effect of congruency only reached significance under unimodal (*p* < .001) and not cross-modal (*p* = .52) conditions. This is in line with the analyses reported in our main manuscript. Unimodal response-incongruent conditions reduced accuracy (*M =* 88.63%, *SE =* .9) compared with congruent (*M =* 92%, *SE =* .73 *p* < .001), stimulus-incongruent (*M =* 93.37%, *SE =* .72 *p* < .001) and neutral conditions (*M =* 91.16%, *SE* = .76, p = .023), whilst accuracy did not significantly differ between unimodal stimulus-incongruent and congruent conditions (*p* = .924). These differences did not occur under cross-modal conditions (respective *p* values under cross-modal conditions were all *p* = 1).

However, the main effect of sensory condition did not reach significance (*F*(1, 102) = .073, *p* = .79, $\eta_{p}^{2}=$ .001) and this was not modulated by age group (*F*(2, 102) = .474, *p* = .62, $\eta_{p}^{2}=$ .009), suggesting similar accuracy between unimodal and cross-modal conditions overall.

|  |  | **Unimodal** | **95% CI**  **lower** | **upper** | **Cross-modal** | **95%CI**  **lower** | **upper** |
| --- | --- | --- | --- | --- | --- | --- | --- |
| Children | C | 87.6 | 85.34 | 89.86 | 89.29 | 86.98 | 91.6 |
|  | SI | 89.19 | 86.96 | 91.41 | 88.49 | 86.01 | 90.97 |
|  | RI | 85.62 | 82.84 | 88.4 | 86.01 | 83.27 | 88.76 |
|  | N | 87.6 | 85.24 | 89.96 | 88.19 | 85.35 | 91.04 |
| Young adults | C | 92.88 | 90.25 | 95.5 | 91.13 | 88.44 | 93.82 |
|  | SI | 92.88 | 90.29 | 95.47 | 89.38 | 86.49 | 92.27 |
|  | RI | 85.62 | 82.38 | 88.85 | 91.26 | 88.07 | 94.46 |
|  | N | 89.79 | 87.03 | 92.54 | 88.04 | 84.73 | 91.34 |
| Older adults | C | 96.88 | 94.29 | 99.46 | 95.7 | 93.06 | 98.35 |
|  | SI | 98.05 | 95.5 | 100.6 | 95.44 | 92.6 | 98.29 |
|  | RI | 94.66 | 91.48 | 97.85 | 95.7 | 92.56 | 98.85 |
|  | N | 96.09 | 93.39 | 98.8 | 96.62 | 93.36 | 99.87 |

Table S3 Accuracy for each age group under congruent (C), stimulus-incongruent (SI), response-incongruent (RI) and neutral (N) conditions under unimodal and cross-modal conditions for experiment 1. Confidence intervals (CI) represent the 95% bootstrapped confidence intervals.

## **S3.2 Experiment 2**

### **S3.2.1 Response times**

Response times for each condition in Experiment 2 are shown in Table S4. A 2 (distractor type; auditory vs. visual) x 4 (congruency; congruent, stimulus-incongruent, response-incongruent and neutral) x 3 (age group) ANOVA showed a main effect of distractor type (*F*(1, 109) = 15.54, *p* < .001, $\eta_{p}^{2}=$ .11) which significantly interacted with age group (*F*(2, 109) = 5.65, *p* = .005, $\eta_{p}^{2}=$.08). Response times were significantly slower with visual (*M* = 874ms, *SE* = 26ms) versus auditory (*M* = 781ms, *SE =* 34ms) distractors. However this difference was only seen in young adults (*p* < .001) and not children (*p* = .72) or older adults (*p* = .12).

There was also a significant main effect of congruency (*F*(2.58, 280.98) = 13.57, *p* < .001, $\eta_{p}^{2}=$ .11), which interacted with age group (*F*(5.16, 280.98) = 3.133, *p* = .008, $\eta_{p}^{2}=$.05). Response times were slower under response interference (*M* = 858ms, *SE* = 31ms) versus congruent (*M* = 783ms, *SE* = 25ms, *p* < .001) and stimulus-incongruent (*M* = 858ms, *SE =* 31ms, *p* = .009) conditions but response times did not significantly differ between response incongruent and neutral (*M* = 841ms, *SE =* 29ms, *p* = 1). Response times were also slower under stimulus-incongruent versus congruent conditions (*p* = .001) but did not significantly differ between stimulus-incongruent and neutral conditions (*p* = 1). As in Experiment 1, and despite presenting no stimuli other than the colour rectangle/word to be identified on neutral trials (compared with the word “Brown” in Experiment 1), response times were significantly slower on neutral versus congruent (*p* < .001). As such the calculation of interference ratios within the main manuscript were based on comparisons between stimulus and response-incongruent conditions with congruent conditions.

Interestingly the effect of congruency interacted with age group because the main effect of congruency reached significance in children (*p* < .001) but not young adults (*p* = .06) or older adults (*p* = .09). However, the effect of congruency was not modulated by distractor type (*F*(2.66, 290.37) = .006, *p* = .35, $\eta_{p}^{2}=$.003) and there was no three-way interaction between congruency, distractor type and age (*F*(5.33, 290.37) = .009, *p* = .72, $\eta^{2}\eta_{p}^{2}=$ .01). This suggests similar congruency effects with visual and auditory cross-modal distractors across age groups.

There was a main effect of age group overall (*F*(2, 109) = 20.28, *p* < .001, $\eta_{p}^{2}=$.28). Children were significantly slower than younger and older adults (*p* < .001 for both comparisons) but response time did not significantly differ between young and older adult groups (*p* = 1).

|  |  | **Unimodal** | **95% CI**  **Lower** | **Upper** | **Cross-modal** | **95%CI**  **Lower** | **Upper** |
| --- | --- | --- | --- | --- | --- | --- | --- |
| Children | C | 961 | 875 | 1048 | 978 | 908 | 1047 |
|  | SI | 1060 | 959 | 1161 | 1082 | 1001 | 1163 |
|  | RI | 1114 | 1005 | 1223 | 1097 | 1018 | 1177 |
|  | N | 1045 | 940 | 1149 | 1072 | 996 | 1148 |
| Young adults | C | 560 | 447 | 674 | 737 | 645 | 829 |
|  | SI | 570 | 437 | 703 | 784 | 678 | 891 |
|  | RI | 610 | 466 | 753 | 793 | 688 | 897 |
|  | N | 587 | 449 | 724 | 809 | 709 | 909 |
| Older adults | C | 698 | 584 | 812 | 762 | 670 | 853 |
|  | SI | 693 | 559 | 826 | 775 | 668 | 882 |
|  | RI | 723 | 580 | 866 | 813 | 709 | 917 |
|  | N | 747 | 609 | 884 | 788 | 688 | 888 |

Table S4 RT for each age group under congruent (C) stimulus-incongruent (SI) response-incongruent (RI) and neutral (N) conditions under unimodal and cross-modal sensory conditions for Experiment 2. Confidence intervals (CI) represent the 95% bootstrapped confidence intervals.

### **S3.2.2 Accuracy**

Accuracy scores for each condition in Experiment 2 are shown in Table S5. A 2 x 4 x 3 ANOVA showed no main effect of distractor type on accuracy (*F*(1, 109) = 2.98, *p* = .09, $\eta_{p}^{2}=$ .03) and this was not influenced by age group (*F*(2, 109) = 1.09, *p* = .34, $\eta_{p}^{2}=$.02). There was a significant main effect of congruency (*F*(2.69, 293.51) = 16.44, *p* < .001, $\eta_{p}^{2}=$ .12) and this interacted with age group (*F*(5.39, 293.51) = 3.51, *p* = .002, $\eta_{p}^{2}=$.05). Accuracy was significantly lower under response-incongruent (*M* = 81.81%, *SE* = 1.15) versus congruent (*M* = 86.28%, *SE* = 0.97, p < .001), stimulus incongruent (*M* = 86.82, *SE* = 0.97, *p* < .001) and neutral (*M* = 86.49, *SE* = 0.98, *p* < .001) conditions. Accuracy under stimulus-incongruent, congruent and neutral conditions did not differ from one another (*p* = 1). Simple main effects showed the effect of congruency only reached significance in children (p < .001) and not young adults (*p* = .017) or older adults (*p* = .45), supporting the conclusion in our main manuscript that children experienced more cross-modal interference than adults

The effect of congruency was not modulated by distractor type (*F*(2.49, 271.24) = .67, *p* = .57, $\eta_{p}^{2}=$.006), suggesting similar congruency effects on accuracy with visual and auditory cross-modal distractors. There was no three-way interaction between congruency, distractor type and age group (*F*(4.98, 271.24) = 2.185, *p* = .044, $\eta_{p}^{2}=$ .04).

|  |  | **Unimodal** | **95% CI**  **lower** | **upper** | **Cross-modal** | **95%CI**  **lower** | **upper** |
| --- | --- | --- | --- | --- | --- | --- | --- |
| Children | C | 80.79 | 77.58 | 84 | 81.31 | 78.09 | 84.54 |
|  | SI | 84.28 | 81.05 | 87.51 | 84.22 | 81.13 | 87.32 |
|  | RI | 73.06 | 68.94 | 77.17 | 76.84 | 73.15 | 80.52 |
|  | N | 80.07 | 76.72 | 83.42 | 84.31 | 81.4 | 87.23 |
| Young adults | C | 86.25 | 82.03 | 90.48 | 88.51 | 84.26 | 92.76 |
|  | SI | 84.32 | 80.06 | 88.57 | 89.45 | 85.37 | 93.52 |
|  | RI | 83.01 | 77.59 | 88.43 | 80.89 | 76.04 | 85.74 |
|  | N | 84.15 | 79.74 | 88.57 | 88.56 | 84.72 | 92.4 |
| Older adults | C | 89.51 | 85.29 | 93.74 | 91.28 | 87.03 | 95.53 |
|  | SI | 89.51 | 85.26 | 93.76 | 89.17 | 85.09 | 93.25 |
|  | RI | 89.13 | 83.71 | 94.55 | 87.92 | 83.07 | 92.76 |
|  | N | 91.75 | 87.33 | 96.16 | 90.11 | 86.27 | 93.95 |

Table S 5 Accuracy for each age group under congruent (C), stimulus-incongruent (SI), response-incongruent (RI) and neutral (N) conditions under unimodal and cross-modal conditions for Experiment 2. Confidence intervals (CI) represent the 95% bootstrapped confidence intervals.

## **S4. Additional tables**

### **S4.1 Full Bayesian Statistics for Comparison of Experiment 1 and 2.**

#### **S4.1.1 Response times: 2 (experiment) x 3 (age group) comparison of general interference on the “ignore auditory” conditions of Experiment 1 and 2**

| Model Comparison – General interference between Experiment 1 and 2 (Response times) | | | | | | | | | | | |
| --- | --- | --- | --- | --- | --- | --- | --- | --- | --- | --- | --- |
| **Models** | | **P(M)** | | **P(M\|data)** | | **BF _M_** | | **BF _01_** | | **error %** | |
| Null model |  | 0.200 |  | 0.237 |  | 1.244 |  | 1.000 |  |  |  |
| Age Group |  | 0.200 |  | 0.346 |  | 2.120 |  | 0.685 |  | 0.030 |  |
| Experiment |  | 0.200 |  | 0.154 |  | 0.729 |  | 1.540 |  | 8.929e -7 |  |
| Age Group + Experiment |  | 0.200 |  | 0.177 |  | 0.859 |  | 1.342 |  | 1.151 |  |
| Age Group + Experiment + Age Group x Experiment |  | 0.200 |  | 0.085 |  | 0.374 |  | 2.775 |  | 2.598 |  |
|  | | | | | | | | | | | |

**Table S6.** 2 (Experiment) x 3 (Age Group) Bayesian ANOVA comparing response time ratios for the “ignore auditory” conditions of Experiment 1 and 2. P(M) =prior model probabilities, P(M|data) =posterior model probabilities. BF _M_  =change from prior to posterior model odds. BF _01_ = Bayes factor for each model against the alternative (favour for the null).

#### **S4.1.2 Accuracy: 2 (experiment) x 3 (age group) comparison of general interference for the “ignore auditory” conditions of Experiment 1 and 2**

| Model Comparison - General interference between Experiment 1 and 2 (Accuracy) | | | | | | | | | | | |
| --- | --- | --- | --- | --- | --- | --- | --- | --- | --- | --- | --- |
| **Models** | | **P(M)** | | **P(M\|data)** | | **BF _M_** | | **BF _01_** | | **error %** | |
| Null model |  | 0.200 |  | 0.417 |  | 2.867 |  | 1.000 |  |  |  |
| Age Group |  | 0.200 |  | 0.310 |  | 1.795 |  | 1.348 |  | 0.027 |  |
| Experiment |  | 0.200 |  | 0.159 |  | 0.755 |  | 2.630 |  | 0.024 |  |
| Age Group + Experiment |  | 0.200 |  | 0.102 |  | 0.454 |  | 4.097 |  | 3.125 |  |
| Age Group + Experiment + Age Group x Experiment |  | 0.200 |  | 0.012 |  | 0.049 |  | 34.338 |  | 2.287 |  |
|  | | | | | | | | | | | |

**Table S7.** 2 (experiment) x 3 (age group) Bayesian ANOVA comparing accuracy ratios for the “ignore auditory” conditions of Experiment 1 and 2. P(M) =prior model probabilities, P(M|data) =posterior model probabilities. BF _M_  =change from prior to posterior model odds. BF _01_ = Bayes factor for each model against the alternative (favour for the null).

#### **S.4.1.1 Response times: 2 (interference type) x 2 (experiment) x 3 (age group) ANOVA**

| Model Comparison- Stimulus and response interference between Experiments 1 and 2 (response times) | | | | | | | | | | | | | | | |  |
| --- | --- | --- | --- | --- | --- | --- | --- | --- | --- | --- | --- | --- | --- | --- | --- | --- |
| **Models** | | **P(M)** | | **P(M\|data)** | | | **BF _M_** | | | **BF _01_** | | | **error %** | | |  |
| Null model (incl. subject) |  | 0.053 |  | | 8.164e -5 |  | | 0.001 |  | | 1.000 |  | |  |  | |
| Interference Type |  | 0.053 |  | | 0.001 |  | | 0.019 |  | | 0.078 |  | | 1.488 |  | |
| Age Group |  | 0.053 |  | | 1.030e -5 |  | | 1.855e -4 |  | | 7.923 |  | | 0.622 |  | |
| Interference Type + Age Group |  | 0.053 |  | | 1.606e -4 |  | | 0.003 |  | | 0.508 |  | | 16.576 |  | |
| Interference Type + Age Group + Interference Type  x  Age Group |  | 0.053 |  | | 0.113 |  | | 2.294 |  | | 7.223e -4 |  | | 2.089 |  | |
| Experiment |  | 0.053 |  | | 1.635e -5 |  | | 2.943e -4 |  | | 4.994 |  | | 2.306 |  | |
| Interference Type + Experiment |  | 0.053 |  | | 2.286e -4 |  | | 0.004 |  | | 0.357 |  | | 11.852 |  | |
| Age Group + Experiment |  | 0.053 |  | | 1.855e -6 |  | | 3.338e -5 |  | | 44.023 |  | | 1.588 |  | |
| Interference Type + Age Group + Experiment |  | 0.053 |  | | 2.412e -5 |  | | 4.341e -4 |  | | 3.386 |  | | 2.494 |  | |
| Interference Type + Age Group + Interference Type  x  Age Group + Experiment |  | 0.053 |  | | 0.020 |  | | 0.367 |  | | 0.004 |  | | 2.667 |  | |
| Interference Type + Experiment+ Interference Type  x  Experiment |  | 0.053 |  | | 0.013 |  | | 0.234 |  | | 0.006 |  | | 4.187 |  | |
| Interference Type + Age Group + Experiment+ Interference Type  x  Experiment |  | 0.053 |  | | 0.002 |  | | 0.028 |  | | 0.052 |  | | 4.672 |  | |
| Interference Type + Age Group + Interference Type  x  Age Group + Experiment+ Interference Type  x  Experiment |  | 0.053 |  | | 0.727 |  | | 47.976 |  | | 1.123e -4 |  | | 5.606 |  | |
| Age Group + Experiment+ Age Group  x  Experiment |  | 0.053 |  | | 1.165e -7 |  | | 2.098e -6 |  | | 700.538 |  | | 2.104 |  | |
| Interference Type + Age Group + Experiment+ Age Group  x  Experiment |  | 0.053 |  | | 1.518e -6 |  | | 2.733e -5 |  | | 53.782 |  | | 2.657 |  | |
| Interference Type + Age Group + Interference Type  x  Age Group + Experiment+ Age Group  x  Experiment |  | 0.053 |  | | 0.001 |  | | 0.022 |  | | 0.068 |  | | 2.703 |  | |
| Interference Type + Age Group + Experiment+ Interference Type  x  Experiment+ Age Group  x  Experiment |  | 0.053 |  | | 1.092e -4 |  | | 0.002 |  | | 0.748 |  | | 13.063 |  | |
| Interference Type + Age Group + Interference Type  x  Age Group + Experiment+ Interference Type  x  Experiment+ Age Group  x  Experiment |  | 0.053 |  | | 0.042 |  | | 0.779 |  | | 0.002 |  | | 2.667 |  | |
| Interference Type + Age Group + Interference Type  x  Age Group + Experiment+ Interference Type  x  Experiment+ Age Group  x  Experiment+ Interference Type  x  Age Group  x  Experiment |  | 0.053 |  | | 0.081 |  | | 1.586 |  | | 0.001 |  | | 4.345 |  | |
|  | | | | | | | | | | | | | | | |  |
| **Table S8.** 2 (interference type) x 2 (experiment) x 3 (age group) Bayesian ANOVA comparing response time ratios for the “ignore auditory” conditions of Experiment 1 and 2. P(M) = prior model probabilities, P(M\|data) = posterior model probabilities. BF _M_  =change from prior to posterior model odds. BF _01_ = Bayes factor for each model against the alternative (favour for the null). | | | | | | | | | | | | | | | |  |

#### **S4.1.2 Accuracy: 2 (interference type) x 2 (experiment) x 3 (age group) ANOVA**

| Model Comparison- Stimulus and response interference between Experiments 1 and 2 (accuracy) | | | | | | | | | | | | | | | | |  |
| --- | --- | --- | --- | --- | --- | --- | --- | --- | --- | --- | --- | --- | --- | --- | --- | --- | --- |
| **Models** | | **P(M)** | | | **P(M\|data)** | | | **BF _M_** | | | **BF _01_** | | | **error %** | | |  |
| Null model (incl. subject) |  | | 0.053 |  | | 8.164e -5 |  | | 0.001 |  | | 1.000 |  | |  |  | |
| Interference Type |  | | 0.053 |  | | 0.001 |  | | 0.019 |  | | 0.078 |  | | 1.488 |  | |
| Age Group |  | | 0.053 |  | | 1.030e -5 |  | | 1.855e -4 |  | | 7.923 |  | | 0.622 |  | |
| Interference Type + Age Group |  | | 0.053 |  | | 1.606e -4 |  | | 0.003 |  | | 0.508 |  | | 16.576 |  | |
| Interference Type + Age Group + Interference Type  x  Age Group |  | | 0.053 |  | | 0.113 |  | | 2.294 |  | | 7.223e -4 |  | | 2.089 |  | |
| Experiment |  | | 0.053 |  | | 1.635e -5 |  | | 2.943e -4 |  | | 4.994 |  | | 2.306 |  | |
| Interference Type + Experiment |  | | 0.053 |  | | 2.286e -4 |  | | 0.004 |  | | 0.357 |  | | 11.852 |  | |
| Age Group + Experiment |  | | 0.053 |  | | 1.855e -6 |  | | 3.338e -5 |  | | 44.023 |  | | 1.588 |  | |
| Interference Type + Age Group + Experiment |  | | 0.053 |  | | 2.412e -5 |  | | 4.341e -4 |  | | 3.386 |  | | 2.494 |  | |
|  |  | |  |  | |  |  | |  |  | |  |  | |  |  | |
| Interference Type + Age Group + Interference Type  x  Age Group + Experiment |  | | 0.053 |  | | 0.020 |  | | 0.367 |  | | 0.004 |  | | 2.667 |  | |
| Interference Type + Experiment+ Interference Type  x  Experiment |  | | 0.053 |  | | 0.013 |  | | 0.234 |  | | 0.006 |  | | 4.187 |  | |
| Interference Type + Age Group + Experiment+ Interference Type  x  Experiment |  | | 0.053 |  | | 0.002 |  | | 0.028 |  | | 0.052 |  | | 4.672 |  | |
| Interference Type + Age Group + Interference Type  x  Age Group + Experiment + Interference Type  x  Experiment |  | | 0.053 |  | | 0.727 |  | | 47.976 |  | | 1.123e -4 |  | | 5.606 |  | |
| Age Group + Experiment+ Age Group  x  Experiment |  | | 0.053 |  | | 1.165e -7 |  | | 2.098e -6 |  | | 700.538 |  | | 2.104 |  | |
| Interference Type + Age Group + Experiment + Age group  x  Experiment |  | | 0.053 |  | | 1.518e -6 |  | | 2.733e -5 |  | | 53.782 |  | | 2.657 |  | |
| Interference Type + Age Group + Interference Type  x  Age Group + Experiment+ Age Group  x  Experiment |  | | 0.053 |  | | 0.001 |  | | 0.022 |  | | 0.068 |  | | 2.703 |  | |
| Interference Type + Age Group + Experiment + Interference Type  x  Experiment + Age Group  x  Experiment |  | | 0.053 |  | | 1.092e -4 |  | | 0.002 |  | | 0.748 |  | | 13.063 |  | |
| Interference Type + Age Group + Interference Type  x  Age Group + Experiment+ Interference Type  x  Experiment + Age group  x  Experiment |  | | 0.053 |  | | 0.042 |  | | 0.779 |  | | 0.002 |  | | 2.667 |  | |
| Interference Type + Age Group + Interference Type  x  Age Group + Experiment + Interference Type  x  Experiment + Age Group  x  Experiment + Interference Type  x  Age Group  x  Experiment |  | | 0.053 |  | | 0.081 |  | | 1.586 |  | | 0.001 |  | | 4.345 |  | |
|  | | | | | | | | | | | | | | | | |  |
| **Table S9.** 2 (interference type) x 2 (experiment) x 3 (age group) Bayesian ANOVA comparing accuracy ratios for the “ignore auditory” conditions of Experiment 1 and 2. P(M) = prior model probabilities, P(M\|data) = posterior model probabilities. BF _M_  = change from prior to posterior model odds. BF _01_ = Bayes factor for each model against the alternative (favour for the null). | | | | | | | | | | | | | | | | |  |

### **S4.2 Consideration of effects in main manuscript in which *p* <= .05**

Within our main manuscript we use a conservative *p* =< .01 to refer to significant effects. However to aid interpretation and ensure we do not miss findings the reader may find of interest we outline the implication of effects in which *p* =< .05 in Table S10 below. Shaded areas indicate effects that are not evident within the main manuscript that may aid interpretation. In summary, these effects tentatively imply a) that stimulus interference might be higher in children and older adults (the former of these did reach significance in Experiment 2) and b) that the facilitatory effect of cross-modal stimulus interference on accuracy increases across development between 6 and 11 years of age.

|  | Analysis | Effect | *p* | Direction of effect and implication |
| --- | --- | --- | --- | --- |
| **General interference comparisons (Experiment 1)** | | | | |
| *Response time* | | | | |
| 1 | 2 (sensory condition) x 3 (age group) ANOVA | Age Group | .05 | Older adults show more interference in general than young adults, whilst there are no other differences between age groups. The interaction with sensory condition reached .01 and shows this arises from unimodal effects. |
| *Accuracy* | | | | |
| 2 | 2 (sensory condition) x 3 (age group) ANOVA | Sensory Condition | .05 | Less accurate for unimodal versus crossmodal. The interaction with interference type reached .01 and shows this arises because of response interference effects. |
| 3 |  | Sensory Condition X Age Group | .03 | Children but not young or old adults show accuracy decrements under cross-modal conditions. Experiment 2 shows that children process cross-modal distractors differently and may make errors with cross-modal incongruency. This is therefore covered in our main manuscript. |
| 4 | Quadratic test | quadratic trend | .02 | Young adults less accurate than children and older adults.  Notably, this may infer a speed accuracy trade off whereby young adults show accuracy and not response time decrements whilst the reverse is the case for children and older adults. However, considering the quadratic trend for stimulus but not response interference (*p* = .02; see below) and the non-significant quadratic trend for response interference on accuracy (*p* = .24; see manuscript) this likely actually reflects facilitation effects of stimulus-interference in childhood and old age that do not occur in adults. |
| **Stimulus and response interference (Experiment 1)** | | | | |
| *Response time* | | | | |
| 5 | 2 (sensory condition) x 2 (interference type) x 3 (age group) ANOVA | Interference Type | .04 | Stimulus interference slowed response times more than response interference. The effects reported suggest in the main manuscript show this arises because of effects in children. |
| 6 | Quadratic test | quadratic trend | .02 | Unimodal stimulus-interference but not response-interference shows a quadratic trend. This suggests stimulus-interference but not response-interference is higher in childhood and old age. |
| *Accuracy* | | | | |
| 7 | 2 (sensory condition) x 2 (interference type) x 3 (age group) ANOVA | Sensory Condition | .04 | Accuracy lower for unimodal versus crossmodal conditions. The interaction with interference type shows this occurs due to response-interference under unimodal but not cross-modal conditions. |
| 8 |  | Interference Type | .02 | Accuracy lower for response-interference. The significant interaction with sensory condition shows that this arises from unimodal effects. |
| 9 |  | Sensory Condition X Age Group | .02 | Children do show accuracy decrements under cross-modal conditions whilst adults do not. This effect was found and discussed in Experiment 2. |
| **Developmental trajectory analysis (Experiment 1)** | | | | |
| *Accuracy* | | | | |
| 10 | 2 (sensory condition) x 2 (interference type) ANCOVA with age as covariate in children. | Sensory Condition X Interference Type | .02 | Difference between stimulus and response interference larger under unimodal versus crossmodal. This is the direction of effects we report throughout and would suggest this emerges with age. |
| 11 |  | Sensory Condition X Interference Type X Age Group | .03 | Under unimodal conditions both SI and RI decrease with age (Figure 4). Under crossmodal conditions accuracy on SI but not RI increases with age.  This suggests cross-modal stimulus-interference increases with development. |
| **Response time distribution analysis (Experiment 1)** | | | | |
| 12 | 2 (sensory condition) x 2 (Interference type) x 9 (percentile) x 3 (age group) ANOVA | Sensory Condition | .03 | Unimodal interference resulted in generally slower response times compared with cross-modal. This effect is reported throughout. |
| **General interference (Experiment 2)** | | | | |
| *Response times* | | | | |
| 13 | 2 (distractor type: auditory, visual) x 3 (age group: children, young adults, older adults) ANOVA. Pair-wise comparison. | children versus older adults | .02 | Children show more cross-modal interference than older adults. |
| 14 | 2 (distractor type) x 2 (interference type) x 3 (age group) ANOVA. Pair-wise comparison. | children versus young adults | .02 | Children showed more cross-modal interference than young adults. |
| **Table S10**. Effects not reported in the main manuscript in which *p* <= .05 and the implication of each of these effects. All non-shaded areas show effects that are accounted for in the main manuscript. Shaded areas show further points the reader may wish to note. | | | | |

###

1. University of Nottingham [↑](#footnote-ref-1)
2. University of St Andrews

   *** Corresponding Author Details**

   Miss Rebecca J. Hirst

   Address:

   School of Psychology

   East Drive

   University Park Campus

   University of Nottingham

   NG7 2RD

   Email: Rebecca.Hirst@nottingham.ac.uk [↑](#footnote-ref-2)
3. Green-house Geisser corrected values. [↑](#footnote-ref-3)
4. Notably, our power analysis was conducted for 3 groups and 4 measures. Therefore we cannot exclude the possibility that a non-significant 3 (age group) x 4 (congruency) x 2 (sensory condition) interaction (3 groups; 8 measures) occurred due to an underpowered sample size. Nevertheless the findings reported here are in line with the adequately powered, non-significant three-way interactions reported in our main manuscript. [↑](#footnote-ref-4)
